# Semi-connected structure and asymmetric gene flow of *Nypa fruticans* Wurmb. across the Philippine archipelago

**DOI:** 10.3389/fpls.2026.1879288

**Published:** 2026-07-06

**Authors:** Rizal M. Suhardi, Junaldo A. Mantiquilla, Piumi Chathurika Palangasinghe, Huie-Chuan Shih, Annamalai Muthusamy, Meng-Shin Shiao, Yu-Chung Chiang

**Affiliations:** 1 Department of Biological Sciences, National Sun Yat-sen University, Kaohsiung, Taiwan; 2 Department of Biological Sciences and Environmental Studies, University of the Philippines Mindanao, Davao City, Philippines; 3 Department of Plant Sciences, The Open University of Sri Lanka, Nugegoda, Sri Lanka; 4 Department of Nursing, Meiho University, Pingtung, Taiwan; 5 Department of Plant Sciences, Manipal School of Life Sciences, Manipal Academy of Higher Education (MAHE), Manipal, Karnataka, India; 6 Research Laboratory Section, Offices of Health Science Research, Faculty of Medicine Ramathibodi Hospital, Mahidol University, Bangkok, Thailand; 7 Department of Biomedical Science and Environment Biology, Kaohsiung Medical University, Kaohsiung, Taiwan

**Keywords:** asymmetric gene flow, microsatellites (SSR), *Nypa fruticans*, Philippines, population structure

## Abstract

The Philippine archipelago is one of the most geographically complex coastal systems in the Indo–West Pacific, comprising numerous islands and semi-enclosed seas linked by narrow straits that together create a highly diverse setting for nearshore exchange. This complex geography contributes to the Philippines’ status as one of the world’s major biodiversity hotspots. *Nypa fruticans* is an ecologically important estuarine mangrove palm in the Philippines that contributes to shoreline stability and supports coastal livelihoods. Its dispersal relies primarily on large, buoyant fruits released directly into coastal waters. Therefore, the genetic diversity and connectivity of populations across different islands of the archipelago remain important questions. We collected 118 individuals from seven coastal regions across Mindanao, Bohol, Luzon, and Palawan to investigate genetic diversity and connectivity across the Philippine archipelago. The populations showed moderate expected heterozygosity (*He* = 0.584–0.719) and consistently lower mean observed heterozygosity (*Ho* = 0.376) than mean expected heterozygosity (*He* = 0.666), resulting in a pervasive heterozygote deficit (*F* = 0.431) and deviations from Hardy–Weinberg equilibrium. This pattern indicated less realised genetic mixing than expected under random mating. Most genetic variance was partitioned among individuals within populations (43%) and within individuals (48%). The results further supported two major genetic clusters (*K* = 2), indicating broad admixture among populations. Directional gene-flow analysis suggested that Palawan acted as a major recipient, whereas western Luzon occupied a key position in the inferred connectivity network. Overall, *N. fruticans* in the Philippines forms a semi-connected metapopulation with modest regional structure and uneven connectivity, suggesting that conserving dispersal pathways and key connectivity nodes, particularly Palawan-linked routes, should be a management priority.

## 1 Introduction

The Philippine archipelago represents one of the most geographically complex coastal systems in the Indo–West Pacific, comprising numerous islands and semi-enclosed seas linked by narrow straits that together form a highly heterogeneous template for nearshore exchange (Gordon et al., 2011; Lermusiaux et al., 2011). Regional circulation is shaped by multiscale processes, including open-ocean boundary currents, inter-basin throughflow, strong tides, and local strait dynamics, resulting in transport pathways that vary markedly among basins and across seasons (Hurlburt et al., 2011; Lermusiaux et al., 2011). In such archipelagic seascapes, dispersal is unlikely to be spatially uniform. Rather, coastline geometry, habitat spacing, and current directionality are expected to generate patchy and anisotropic exchange, including asymmetric connectivity and a limited number of high-probability routes linking populations (Riginos et al., 2016; Selkoe et al., 2016; Jahnke and Jonsson, 2022). Consistent with this expectation, connectivity analyses in the Philippines highlight basin–strait structure and seasonally varying circulation as key drivers of asymmetric dispersal and partial barriers to exchange among coastal populations (Pata and Yñiguez, 2021). The complex geographic seascape leads to an interesting question: whether the populations of a species in the Philippines showed a strongly differentiated genetic structures or a semi-connected population structure marked by partial exchange among some populations but restricted connectivity across major geographic barriers.

*Nypa fruticans* is a valuable model for testing how coastal geography influences genetic connectivity in the Philippines, as its distribution and dispersal are tightly associated with nearshore and estuarine settings. The species is largely confined to brackish estuarine habitats shaped by tidal processes (Tomlinson, 2016; Mantiquilla et al., 2022). Dispersal success depends on both transport along the coastal interface and subsequent establishment in suitable estuarine environments (Tomlinson, 2016; Mantiquilla et al., 2022). Moreover, because *N. fruticans* disperses mainly via large, buoyant fruits released into coastal waters, realized movement is strongly influenced by tidal dynamics, nearshore circulation, and coastline geometry (Das and Ghose, 2003; Bobrov et al., 2012). As in other mangroves, the interaction between hydrodynamic and geomorphological factors plays important roles in shaping the genetic structures of populations (Van der Stocken et al., 2019). Comparative studies across Southeast Asian mangroves further indicate that genetic differentiation across coastal and biogeographic barriers often varies among species according to dispersal capacity and propagule traits (Wee et al., 2020). Together, this evidence indicates that *N. fruticans* is a suitable model to study how heterogeneous coastal circulation translates into genetic connectivity across an archipelagic landscape.

Previous genetic studies of *N. fruticans* have documented substantial variation in genetic diversity and population differentiation across its Indo–West Pacific range. The studies showed that the species can exhibit both genetically depauperate, sometimes clonal, range-edge populations and regionally differentiated population groups shaped by founder effects and limited gene flow (Jian et al., 2010; Tsuji et al., 2016; Mantiquilla et al., 2022). A chromosome-level genome assembly for *N. fruticans* has recently confirmed a low mutation rate and strong purifying selection in this lineage (Wu et al., 2024). Additionally, seascape genetic research on mangroves, seagrasses, and other coastal taxa demonstrated that current-driven connectivity can explain genetic structure better than geographic distance alone and can generate asymmetric exchange among regions (Hernawan et al., 2017; Geng et al., 2021; Gouvêa et al., 2023). However, population connectivity of *N. fruticans* in the Philippine archipelago remains an interesting question. In previous study, Philippine populations of *N. fruticans* were collectively treated as a single regional unit in a broader Indo–West Pacific comparison, without resolving within-archipelago structure (Mantiquilla et al., 2022). As a result, it remains unclear whether populations of Philippine archipelago are broadly panmictic, highly fragmented across islands and basins, or only partially connected via a limited number of directional dispersal corridors. This distinction is particularly significant in the Philippines, where semi-enclosed seas and straits generate highly heterogeneous and seasonal circulation that is expected to drive asymmetric dispersal routes among coastal regions.

Despite increasing interest in seascape genetics of coastal plants, no study has explicitly examined population connectivity and directional gene flow of *Nypa fruticans* within the Philippine archipelago. Previous studies primarily focused on broad Indo–West Pacific comparisons and treated Philippine populations as a single regional unit, preventing the detection of within-archipelago genetic structure and connectivity patterns. Consequently, it remains unknown whether Philippine populations form a panmictic system, a strongly fragmented network, or an intermediate semi-connected metapopulation maintained by asymmetric dispersal pathways. Addressing this gap is essential for understanding how complex archipelagic seascapes influence genetic connectivity in estuarine mangrove species.

In this study, we examined genetic diversity, population structure, and gene flow of *N. fruticans* across seven coastal regions of the Philippines. Specifically, we tested the hypothesis that *N. fruticans* populations across the Philippines exhibited a semi-connected structure in which connectivity was non-uniform and maintained by asymmetric gene flow along dominant oceanographic routes. Here, we use “semi-connected” to refer to a metapopulation structure in which populations are linked by limited and uneven dispersal pathways rather than uniform gene flow. Using this framework, we assessed whether the genetic architecture of this estuarine specialist reflects the fragmented geography and directional hydrodynamic forcing of the Philippine archipelago.

## 2 Materials and methods

### 2.1 Field sampling and tissue preservation

**Figure 1:**
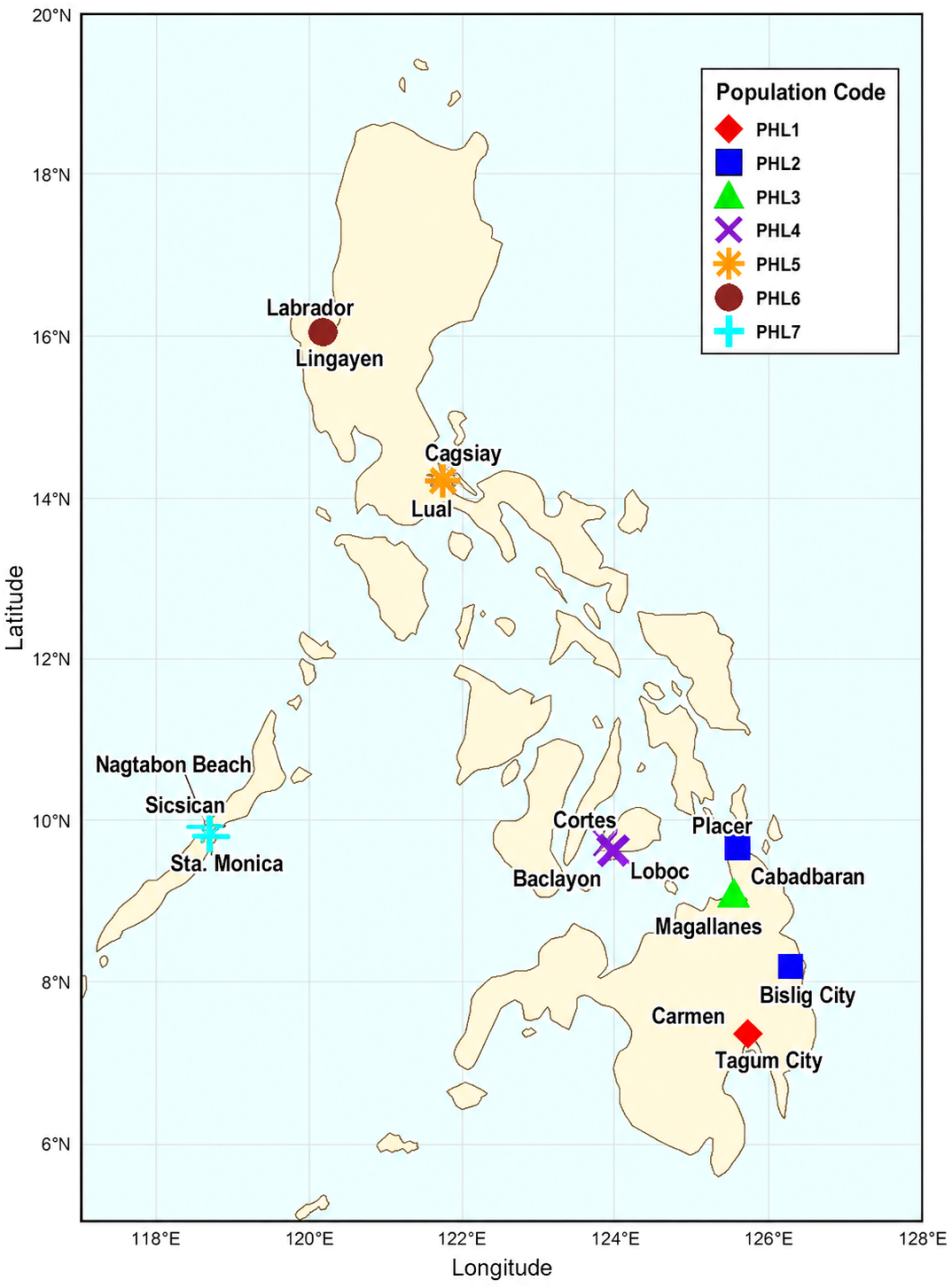
Sampling sites and regional populations of *Nypa fruticans* in the Philippines. The map shows the locations of 16 sampling sites across the Philippine archipelago. Sampling sites located within the same river–estuary system and adjacent coastal sea were pooled into seven regional populations (PHL1–PHL7) for population genetic analyses. Colors indicate regional population assignments, and site codes correspond to those listed in **Table 1**.

A total of 118 individuals were sampled from 16 sampling sites distributed across the Philippine archipelago (**Table 1**; **Figure 1**). For population-genetic analyses, sampling sites were grouped *a priori* into seven regional population units based on geographic proximity, shared river-estuary systems, and expected propagule exchange within the same nearshore circulation setting. This classification was established before genetic analyses to avoid circular inference and was intended to represent biologically meaningful regional dispersal units rather than genetically defined clusters. We acknowledge that finer-scale genetic subdivision may exist within some regional units, and future studies with denser sampling across individual estuaries will be useful for evaluating within-region population structure.

**Table 1:** Sampling sites of *Nypa fruticans* in the Philippines.

Location (code of population)		Latitudes	Longitudes	Samples	
1.Mindanao-South (PHL1)	Carmen	N 7°21’44.6”	E 125°42’30.3”	15	
	Tagum City	N 7°21’34.4”	E 125°46’10.9”		
2. Mindanao-East (PHL2)	Bislig City	N 8°10’27.6”	E 126°17’20.9”	24	
	Placer	N 9°38’51.1”	E 125°36’14.8”		
3. Agusan Basin (PHL3)	Cabadbaran	N 9°04’03.9”	E 125°32’09.3”	12	
	Magallanes	N 9°01’33.5”	E 125°30’52.7”		
4. Central (PHL4)	Baclayon	N 9°36’52.9”	E 123°56’14.3”	19	
	Cortes	N 9°43’20.6”	E 123°52’36.2”		
	Loboc	N 9°37’18.9”	E 124°1’25.7”		
5. Luzon-East (PHL5)	Cagsiay	N 14°15’41.1”	E 121°44’18.3”	15	
	Lual	N 14°11’09.4”	E 121°43’40.3”		
6. Luzon-West (PHL6)	Labrador	N 16°01’04.4”	E 120°09’06.8”	18	
	Lingayen	N 16°00’29.1”	E 120°12’53.5”		
7. Palawan (PHL7)	Nagtabon Beach	N 9°55’54.5”	E 118°38’32.5”	15	
	Sta. Monica	N 9°47’54.3”	E 118°43’42.1”		
	Sicsican	N 9°47’52.2”	E 118°43’20.9”		
Total					118

Sixteen sampling sites were pooled into seven regional populations (PHL1–PHL7) for population genetic analyses. Sample size (N) represents the total number of individuals per regional population.

The resulting regional units span major biogeographic sectors of the archipelago, including southern and eastern Mindanao (including the Agusan basin), central Visayas (Bohol), eastern and western Luzon, and Palawan. Sampling covered contrasting ecological settings within these regions, ranging from dense stands in regularly flooded brackish estuaries (e.g., Lingayen, Pangasinan; Cortes, Bohol) to more open coastal habitats (e.g., Placer, Surigao del Norte; Nagtabon Beach, Palawan) and seasonally flooded upstream zones (e.g., Carmen, Davao del Norte). Because *N. fruticans* spreads clonally via horizontally growing, dichotomously branching rhizomes that often form dense monospecific stands, meticulous care was taken to minimize the probability of resampling the same genet. Following approaches commonly used in mangrove population genetic studies (Ngeve et al., 2016; Geng et al., 2021), putative genets were operationally defined as visually discontinuous rhizome clusters within each sampling site. Only one ramet was sampled per cluster, and a minimum inter-sample distance of approximately 10 m was maintained within each site. This conservative spacing reduces the likelihood that multiple sampled individuals belong to the same genet and thereby enhances the reliability of individual-based genetic inferences, despite constraining the total sample size per locality in smaller estuarine systems.

Samples were obtained from the young leaves of individual plants. From each leaf, midrib-free segments from the central leaflets were trimmed to approximately 10–15 cm, placed in porous paper envelopes or mesh sachets, and rapidly desiccated in sealed bags containing silica gel at an approximate sample-to-desiccant ratio of 1:10. Dried leaf tissues were transported to the Department of Biological Sciences, National Sun Yat-sen University (Kaohsiung, Taiwan) and stored at room temperature until DNA extraction.

### 2.2 DNA extraction and microsatellite genotyping

Total genomic DNA was isolated from silica-gel dried leaf tissues using the RBC Real Genomics™ Box YGP 100 Kit (RBC Biosciences, New Taipei City, Taiwan), following the manufacturer’s protocol with minor modifications optimized for *N. fruticans*. DNA quality and concentration were assessed using a Nano-300 Micro-spectrophotometer (Allsheng, Hangzhou, China). Consistent with concentration standards used in previous genetic studies of *Nypa* (Mantiquilla et al., 2022), DNA samples were diluted to a final working concentration of 5 ng/μL and stored at –20 °C until PCR amplification.

A set of 18 polymorphic microsatellite (SSR) markers previously developed for *N. fruticans* by Mantiquilla et al. (2021), was used for genotyping (**Supplementary Table 1**). Primer development, fluorescent labeling, fragment analysis procedures, and genotyping protocols are described in detail in the original publication (Mantiquilla et al., 2021); the present study utilized the resulting microsatellite dataset for downstream population genetic analyses. Polymerase Chain Reactions (PCR) were performed in a final reaction volume of 20 μL. Each PCR reaction contained 10.6 μL of nuclease-free water, 2.0 μL of 10× PCR buffer (final concentration 1×), 2.0 μL of dNTP mixture, 2.0 μL of forward and reverse primer mix, 0.4 μL of bovine serum albumin (BSA), 0.5 μL of Taq DNA polymerase, and 0.5 μL of genomic DNA template (approximately 2.5 ng). Amplifications were performed using a Multigene Optimax Thermal Cycler (Labnet International) under the following conditions: initial denaturation at 94 °C for 2 mins; followed by 35 cycles of denaturation at 94 °C for 45 s, primer annealing at locus-specific temperatures ranging from 50-60 °C for 45 s (optimized conditions for each locus are listed in **Supplementary Table 1**), and extension at 72 °C for 50 s; with a final extension step at 72 °C for 7 min. PCR products were initially verified by electrophoresis on 1.5% agarose gels.

### 2.3 Null allele estimation and locus filtering

Null allele frequencies were estimated for each locus and population using the estimator based on heterozygote deficits (Brookfield, 1996). To diagnose potential genotyping artifacts and minimize their influence on downstream analyses, null allele frequencies were analyzed in R (R Core Team, 2023) using the packages of adegenet (Jombart, 2008), PopGenReport (Adamack and Gruber, 2014), and poppr (Kamvar et al., 2014), with inference based on 1,000 bootstrap iterations (Brookfield, 1996). Several loci exhibited consistently high null allele frequencies across populations, whereas most loci showed low to moderate values. To reduce bias in downstream estimates of heterozygosity and population differentiation, loci with mean null allele frequencies greater than 0.50 across populations, together with repeatedly high estimates in multiple populations, were treated as problematic and excluded from subsequent analyses. Applying this criterion to the Philippine dataset identified six loci (Nypa4, Nypa6, Nypa11, Nypa12, Nypa13, and Nypa18) for removal. The remaining 12 loci constituted the filtered dataset used in all subsequent analyses (**Supplementary Table 2**).

### 2.4 Genetic diversity and analysis

Microsatellite genotypes were formatted in GenAlEx v6.5 (Peakall and Smouse, 2012). For each population, genetic diversity indices were calculated, including number of alleles (*N*_a_), effective number of alleles (*N*_e_), Shannon’s information index (I), observed heterozygosity (*H*_o_), expected heterozygosity (*H*_e_), unbiased expected heterozygosity (u*H*_e_), and the inbreeding coefficient (F). Deviations from Hardy–Weinberg equilibrium (HWE) were tested for each locus–population combination using chi-square tests in GenAlEx. To evaluate whether HWE deviations were consistent with locus-specific genotyping artifacts (e.g., null alleles) rather than population-level processes, the pattern and consistency of HWE deviations across populations were examined for each locus. The number of private alleles was determined in GenAlEx v6.5 (Peakall and Smouse, 2012) and the number of rare alleles was computed in R (R Core Team, 2023) using the adegenet and poppr packages (Jombart, 2008; Kamvar et al., 2014). Both metrics were tabulated per population to assess population distinctiveness.

### 2.5 Population differentiation and structure

Genetic differentiation among populations was quantified using analysis of molecular variance (AMOVA) in GenAlEx v6.5 (Peakall and Smouse, 2012), with significance assessed using 999 permutations and statistical significance declared at *p* < 0.001. AMOVA partitioned variance among populations, among individuals within populations, and within individuals. Pairwise *F*_ST_ values were calculated following Weir and Cockerham (1984).

To visualize genetic relationships among individuals without imposing additional grouping assumptions, PCoA was performed in GenAlEx based on a codominant genotypic distance matrix (Peakall and Smouse, 2012). Complementary to PCoA, discriminant analysis of principal components (DAPC) was performed in R using adegenet (Jombart et al., 2010) on the filtered 12-locus dataset, with individuals grouped by sampling population. DAPC provides a multivariate ordination that maximizes among-group variance while minimizing within-group variance. The number of retained principal components was determined using cross-validation as implemented in adegenet. Inertia ellipses were used to represent the dispersion of each population in discriminant space. Because DAPC uses predefined population assignments and is designed to maximize among-group variance, its results were interpreted as a complementary visualization of genetic relationships among regional units rather than as an independent test of population structure; inferences regarding differentiation therefore rest primarily on PCoA, STRUCTURE, and AMOVA.

Bayesian clustering analyses were carried out using STRUCTURE v2.3.4 (Pritchard et al., 2000) under an admixture model with correlated allele frequencies. For each hypothesized number of clusters (K), 10 independent runs were performed, each with 100,000 burn-in iterations followed by 1,000,000 MCMC iterations to ensure convergence and adequate sampling of the posterior distribution. The most likely K was determined using the ΔK method (Evanno et al., 2005), and calculated using the web-based tool Structure Selector (Li and Liu, 2018). Replicate runs were aligned and summarized across K values using CLUMPP (Jakobsson and Rosenberg, 2007) and Clumpak (Kopelman et al., 2015) to account for label switching and to identify the major clustering mode. Consistency among replicate runs was assessed to ensure stable clustering solutions prior to determining the most likely K.

### 2.6 Gene flow and migration network

Directional gene flow among populations was inferred using the web-based application divMigrate-online at https://popgen.shinyapps.io/divMigrate-online/ (Sundqvist et al., 2016) based primarily on Jost’s D (Jost, 2008), which provides a robust measure of genetic differentiation for highly polymorphic loci. Migration networks were estimated using 1,000 bootstrap replicates, and only statistically significant migration pathways (α = 0.05) were retained. In addition, a filter threshold of 0.20 was applied to visualize only the strongest directional migration links and to reduce network complexity. To assess the sensitivity of the directional network to the threshold choice, the analysis was repeated without a threshold (i.e., a filter of 0). The resulting network topology was identical to that obtained at the 0.20 threshold, confirming that the threshold did not artificially introduce or suppress any directional pathways.

Contemporary effective population size (*N*_e_) was estimated for each regional population using the linkage disequilibrium method (Hill, 1981; Waples and Do, 2008) implemented in NeEstimator v2.1 (Do et al., 2014), with 95% confidence intervals derived parametric method. As a complementary, frequency-based measure of historical connectivity, the effective number of migrants per generation (*Nm*) was estimated using the private-allele method (Slatkin, 1985; Mantiquilla et al., 2022), with correction for sample size. This approach uses the frequency of private alleles among populations to infer gene flow under drift–migration equilibrium.

To identify microsatellite loci potentially under selection, a genome-scan was performed using BayeScan v2.01 (Foll and Gaggiotti, 2008), which detects candidate outlier loci from differences in allele frequencies among populations. The analysis used 20 pilot runs of 5,000 iterations, a burn-in of 50,000, and 100,000 total MCMC iterations with a thinning interval of 10. Loci were identified as outliers at a false discovery rate (FDR) of 0.05. A positive locus-specific alpha (α) indicates diversifying (directional) selection, whereas a negative value is consistent with balancing selection. This step was used to confirm that the retained loci behaved as approximately neutral markers prior to population genetic inference.

## 3 Results

### 3.1 Genetic diversity and allelic richness

Microsatellite loci are susceptible to null alleles because mutations in primer-binding regions can prevent successful amplification of certain alleles. As a result, heterozygous individuals may be incorrectly scored as homozygotes, potentially biasing estimates of genetic diversity, heterozygosity, and population differentiation. To minimize these effects, null allele frequencies were evaluated for all loci, and loci exhibiting consistently high frequencies were excluded from downstream analyses. A conservative threshold was adopted based on empirical studies and simulations, which indicated that null allele frequencies below ~0.20 generally have limited impact on assignment tests and many population genetic inferences (Dakin and Avise, 2004; Carlsson, 2008), whereas higher values may introduce significant bias in heterozygosity and *F* statistics if not corrected (Chapuis and Estoup, 2007). Several loci exhibited consistently high null allele frequencies (≥ 0.50 in multiple populations), whereas most loci showed low to moderate values. Locus-wise estimates of mean null allele frequency across populations identified six loci with the highest values —Nypa4 (0.903), Nypa6 (0.636), Nypa11 (0.817), Nypa12 (0.662), Nypa13 (0.508), and Nypa18 (1.000) — supporting their exclusion from downstream analyses (**Supplementary Table 2**). These loci may be influenced by genotyping artifacts (e.g., allele dropout or primer mismatch), which can contribute to heterozygote deficits and inflated *F*_IS_. In contrast, several loci (e.g., Nypa9 and Nypa14) showed low pooled null-allele estimates, suggesting comparatively reliable genotyping performance in this dataset. The remaining 12 loci formed a filtered dataset, which was used in all subsequent analyses in this study. The raw microsatellite genotype data used for the final analyses, after locus filtering, are provided in **Supplementary Raw Data Sheet 1**.

All seven regional populations were polymorphic across the 12 microsatellite loci and exhibited moderate levels of genetic diversity (**Table 2**). The mean number of alleles per locus (*N*_a_) ranged from 4.250 to 6.083 in each population with an average of 5.179 across populations. This indicated that multiple alleles were segregated at most loci rather than being restricted to one or two variants. Among populations, Palawan (PHL7) showed the highest number of alleles (*N*_a_) and effective number of alleles (*N*_e_), whereas Luzon-West (PHL6) showed lowest *N_a_* and *N_e_* among all the populations. Expected heterozygosity (*H*_e_) was moderate across populations, whereas observed heterozygosity (*H*_o_) was consistently lower than *H*_e_. This resulted in positive inbreeding coefficients (*F*_IS_) in all populations, indicating a widespread heterozygote deficit across the archipelago.

**Table 2:** Genetic diversity indices of seven regional populations of *Nypa fruticans* based on the filtered 12-locus microsatellite dataset.

Populations		N	Na	Ne	I	Ho	He	uHe	F	%P
PHL1	Mean	15.000	4.750	3.109	1.235	0.311	0.636	0.657	0.511	100
	SE	0.000	0.538	0.330	0.113	0.068	0.038	0.039	0.100	
PHL2	Mean	23.250	6.000	3.512	1.403	0.361	0.692	0.707	0.484	100
	SE	0.351	0.444	0.306	0.083	0.055	0.025	0.026	0.068	
PHL3	Mean	12.000	4.250	3.003	1.158	0.375	0.622	0.649	0.380	100
	SE	0.000	0.463	0.356	0.106	0.071	0.038	0.040	0.115	
PHL4	Mean	18.917	5.667	3.870	1.449	0.384	0.719	0.739	0.444	100
	SE	0.083	0.432	0.326	0.087	0.055	0.026	0.027	0.098	
PHL5	Mean	15.000	5.250	3.784	1.382	0.394	0.698	0.722	0.455	100
	SE	0.000	0.566	0.418	0.115	0.077	0.034	0.035	0.095	
PHL6	Mean	18.000	4.250	2.913	1.111	0.329	0.584	0.601	0.420	100
	SE	0.000	0.566	0.346	0.138	0.070	0.058	0.060	0.123	
PHL7	Mean	15.000	6.083	3.916	1.492	0.478	0.712	0.736	0.324	100
	SE	0.000	0.596	0.410	0.107	0.038	0.031	0.032	0.049	
Total	Mean	16.738	5.179	3.444	1.318	0.376	0.666	0.687	0.431	100
	SE	0.375	0.205	0.137	0.042	0.024	0.015	0.015	0.035	

N, sample size; N_a_, number of alleles; N_e_, number of effective alleles; I, Shannon’s Information Index; H_o_, observed heterozygosity; H_e_, expected heterozygosity, uH_e_, unbiased expected heterozygosity; F: fixation index, %P, percent polymorphic loci; SE, standard error.

*F* represents the population-level inbreeding coefficient estimated from heterozygote deficiency and is provided as a descriptive index. The statistical significance of heterozygote deficits was evaluated using locus–population Hardy–Weinberg equilibrium tests, with results provided in **Supplementary Table 3**.

Although population-level F values in **Table 2** are presented as descriptive estimates, the statistical support for heterozygote deficits was evaluated using locus–population Hardy–Weinberg equilibrium tests. These tests indicated significant departures from Hardy–Weinberg equilibrium (HWE) in most locus–population combinations (P < 0.05). Although the magnitude of the deficit varied among populations, the qualitative pattern was consistent across the archipelago, from Mindanao (PHL1–PHL3) through Central Visayas (PHL4) to Luzon (PHL5–PHL6) and Palawan (PHL7). Non-significant results were rare and restricted to a small subset of loci in specific populations, such as Nypa9 in PHL4 and Nypa17 in PHL2 (**Supplementary Table 3**).

### 3.2 Population structure and differentiation

**Figure 2:**
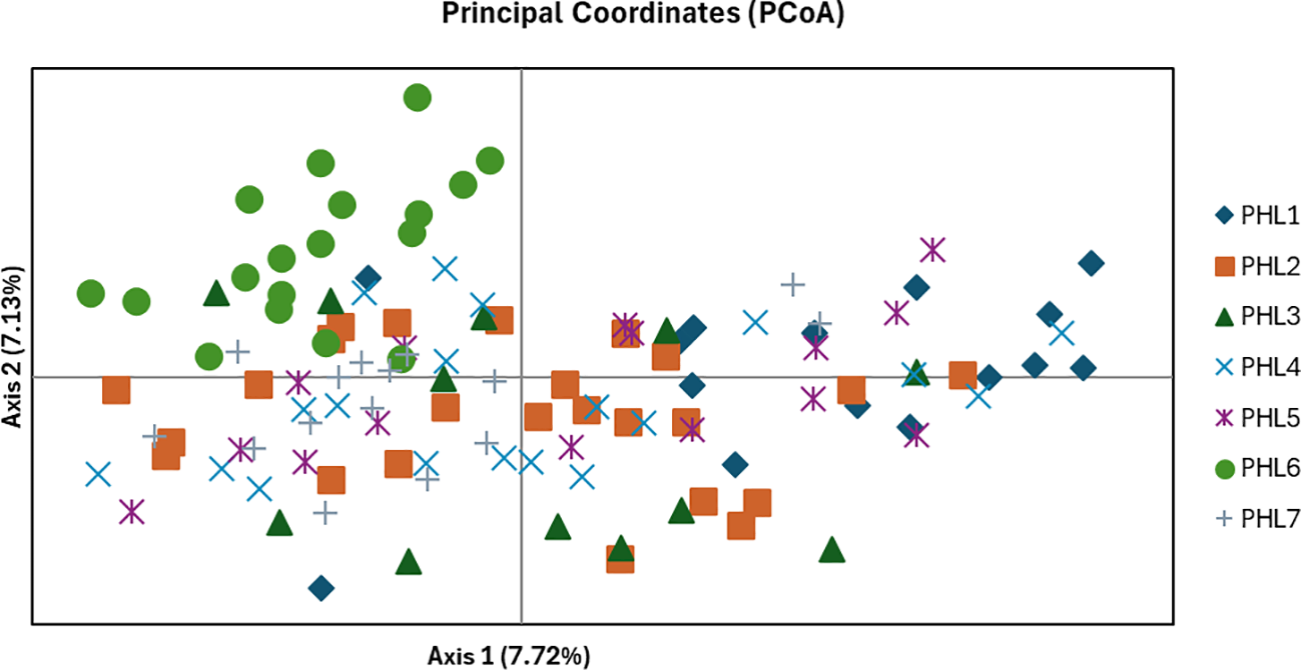
Principal coordinate analysis (PCoA) of *N. fruticans* populations based on codominant genotypic distances. Each point represents an individual, colored by regional population (PHL1–PHL7). The first two coordinates explain 7.72% and 7.13% of the total genetic variation, respectively.

Analysis of molecular variance (AMOVA) indicated that most genetic variation resided within individuals (48%) and among individuals within populations (43%), whereas only 9% of the total variation was partitioned among populations (**Table 3**). This among-population component corresponded to a global *F*_ST_ value of 0.086 (p < 0.001, 999 permutations), indicating weak but detectable population genetic differentiation at the regional scale. These results suggest that substantial genetic variation is maintained within populations despite the presence of modest genetic structuring among regions. The PCoA based on codominant genotypic distances showed partial, but incomplete, clustering of individuals in the seven regional populations. In particular, PHL6 showed a modest displacement relative to the other populations along Coordinate 2 (**Figure 2**). In contrast, the remaining six populations (PHL1, PHL2, PHL3, PHL4, PHL5, and PHL7) showed substantial overlap in ordination space, with individuals largely intermingled in the central and right portions of the plot and no clearly separated clusters (**Figure 2**). Although PHL2 is geographically distant from PHL1 and PHL3 along the eastern Mindanao coastline, its position in the ordination largely overlaps with these populations. This pattern suggests limited differentiation in multilocus genotype composition at the scale captured by the first two PCoA axes and may reflect coastal current-mediated connectivity rather than simple isolation-by-distance.

**Table 3:** Analysis of molecular variance (AMOVA) among seven regional populations of *Nypa fruticans* based on the filtered 12-locus dataset.

Source	df	SS	MS	Est. var.	%	F-Statistics	p-value
Among populations	6	117.042	19.507	0.398	9%	FST = 0.086	0.001
Among individuals	111	688.958	6.207	1.987	43%	FIS = 0.471	0.001
Within individuals	118	263.500	2.233	2.233	48%	FIT = 0.516	0.001
Total	235	1069.500		4.617	100%		

df, Degrees of Freedom; SS, Sum of squares; MS, Mean Square; Est. Var., Estimated Variance.

**Figure 3:**
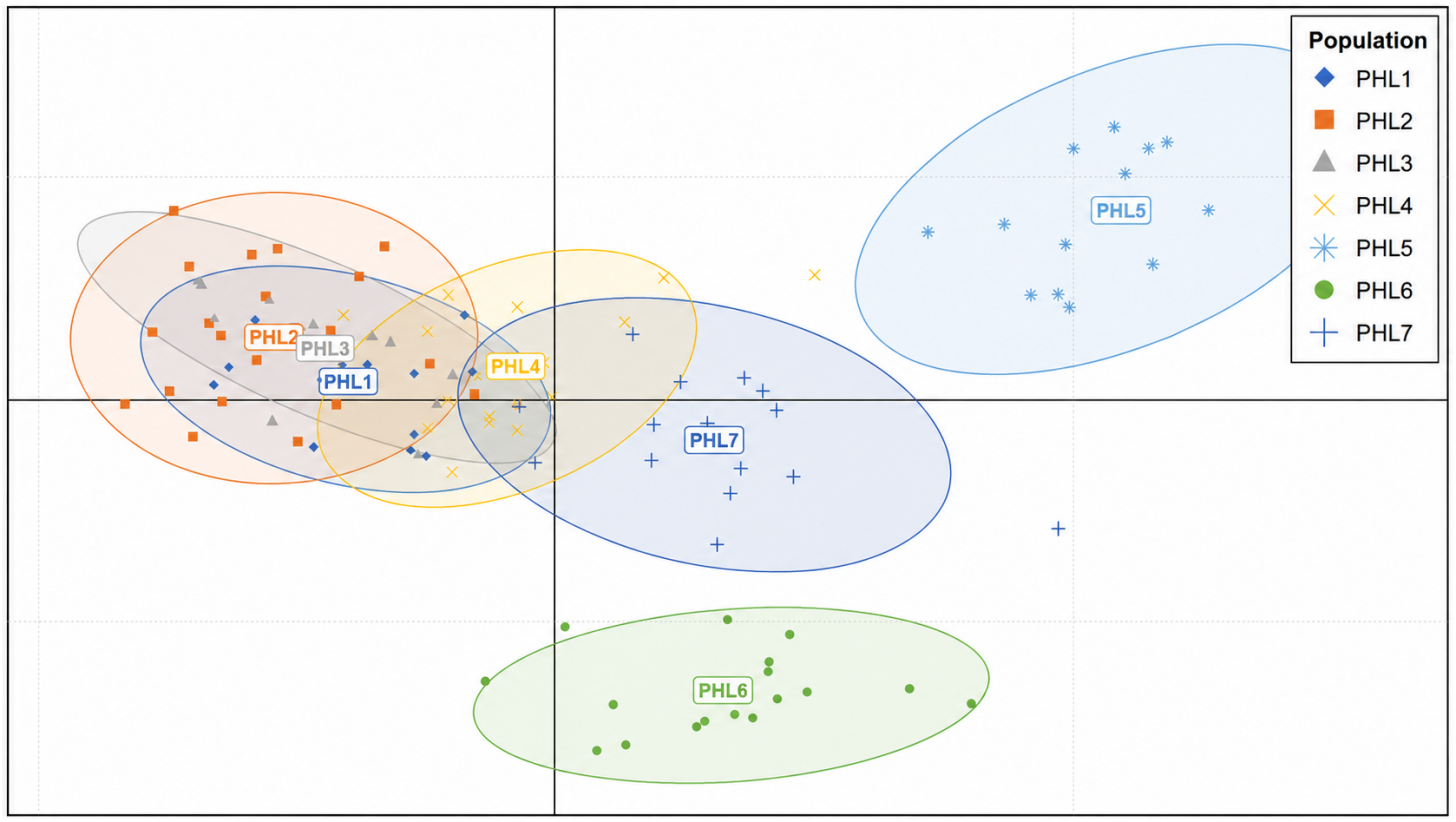
Discriminant analysis of principal components (DAPC) of *N. fruticans* populations. Individuals are grouped by regional population (PHL1–PHL7). The number of retained principal components was determined by cross-validation. Inertia ellipses represent the dispersion of each population in discriminant space.

The discriminant analysis of principal components (DAPC) further refined patterns of population structure by maximizing separation among predefined population groups (**Figure 3**). The results indicated that four clusters can be identified: (cluster 1) PHL1, PHL2, PHL3, and PHL4; (cluster 2) PHL7 with partial overlap with cluster (cluster 1); (cluster 3) PHL5; (cluster 4) PHL6. However, substantial overlap among ellipses was evident within cluster 1, particularly among the Mindanao populations (PHL1–PHL3) together with PHL2 and PHL4, suggesting weak differentiation among these regional units. In contrast, PHL5 and PHL6 formed well-separated groups with minimal overlap, whereas PHL7 occupied a shifted position in discriminant space with partial overlap mainly with PHL4. Overall, DAPC supported the presence of modest but detectable population differentiation superimposed on a largely shared genetic background.

**Figure 4:**
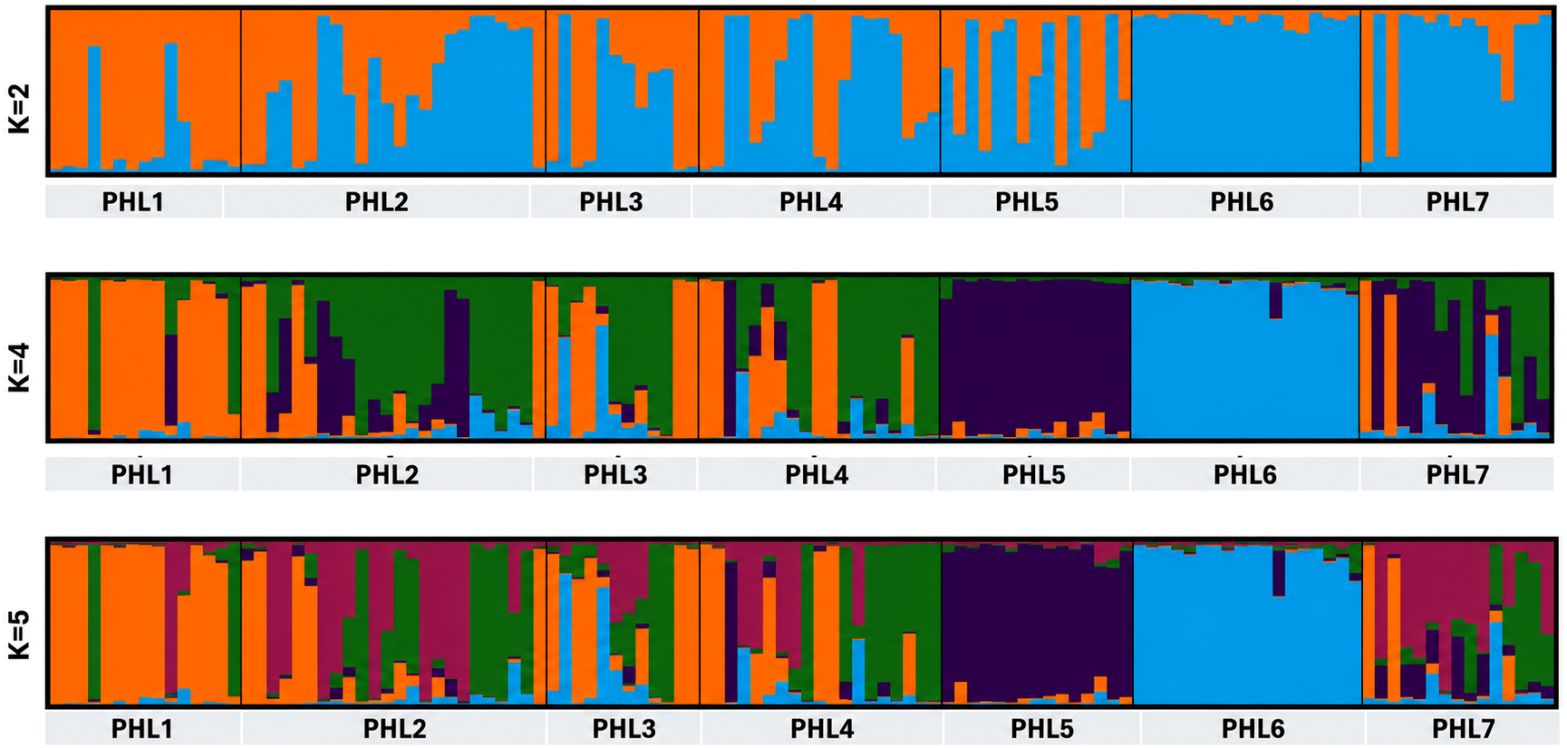
Bayesian clustering results from STRUCTURE analysis of *N. fruticans* populations. Results are shown for K = 2, K = 4, and K = 5 under an admixture model. Each vertical bar represents an individual, and colors indicate the inferred ancestry clusters. The optimal number of clusters (K = 2) and the secondary sub-structuring partitions (K = 4 and K = 5) were determined using the Evanno Delta K method, presented here in numerical order for visual readability.

Bayesian clustering analysis (STRUCTURE) further supported the presence of low-to-moderate population structure (**Figure 4**). Based on the Evanno method, K = 2 was identified as the primary and most optimal number of genetic clusters, exhibiting a dominant peak with the highest delta K value (Delta K = 183.30). To explore finer-scale hierarchical structures and alternative configurations, the partitions for K = 5 (Delta K = 41.36) and K = 4 (Delta K = 23.41) were also evaluated in numerical order for structural readability (**Supplementary Table 4**). K-selection statistics (ΔK, mean LnP(K), and run convergence assessed by CLUMPP) are provided in **Supplementary Figure 1**. At K = 2, all seven populations showed admixture between the two ancestry clusters: a gradual increase of one cluster (orange) and decrease of another cluster (blue) from population PHL1 to PHL7 was observed, with PHL4 showed an intermediate pattern among all populations (**Figure 4**).

When looking at higher K values, finer genetic subdivisions emerged. At K = 4, two additional distinct genetic components were resolved: a third cluster (green) predominant in populations PHL1 to PHL4, and a fourth cluster (purple) that uniquely characterized the majority of individuals in population PHL5. At K = 5, further sub-structuring was observed with the appearance of a fifth cluster (maroon) that was primarily restricted to populations PHL1, PHL2, and PHL7. Overall, these multi- K bar plots reveal a hierarchically nested genetic structure, demonstrating that the higher clusters (K = 4 and K = 5) refine rather than contradict the primary dual-cluster split established at K = 2.

### 3.3 Gene flow and migration patterns

Private allele-based estimates indicated intermediate levels of gene flow among the populations of the Philippines (**Table 4**). The sample-size corrected estimate of the effective number of migrants per generation was *Nm* = 1.410, indicating that population exchange has been sufficient to maintain broad genetic connectivity across the archipelago, but not strong enough to eliminate regional differentiation entirely. This interpretation is consistent with the AMOVA result showing that 9% of total genetic variation was partitioned among populations (*F*_ST_ = 0.086), as well as with the partial overlap and admixture detected in the PCoA, DAPC, and STRUCTURE analyses.

**Table 4:** Private allele–based estimates of the effective number of migrants per generation (*Nm*) among seven regional populations of *N. fruticans*.

Sample size	Nm
Mean N = 10	2.360
Mean N = 25	1.098
Mean N = 50	0.750
Corrected value for sample size	1.410
Number of populations detected: 7 Number of loci detected: 12	
Mean sample size: 16.738	
Mean frequency of private alleles, p (1): 0.074	

*Nm* values are shown for standardized mean sample sizes (N = 10, 25, and 50) following the private-allele method, together with the sample-size corrected estimate used as the primary summary of historical gene flow.

Private allele counts varied among the seven regional populations (**Supplementary Table 5**). PHL7 (Palawan) harbored the highest number of private alleles (n = 11, whereas PHL1 contained none. In addition to rare alleles (frequency < 0.05) showed a comparable distribution among populations, with PHL7 (Palawan) again harboring the highest count (n = 22), followed by PHL2 (n = 20), whereas PHL3 contained the fewest (n = 5; **Supplementary Table 5**). Contemporary effective population size estimates were uniformly small across all populations (*N*_e_ = 12.1–32.3; **Supplementary Table 6**), with PHL3 recording the lowest estimate (*N*_e_ = 12.1; 95% CI: 5.8–34.3) and PHL2 the highest (*N*_e_ = 32.3; 95% CI: 20.0–66.5). These low *N*_e_ values are consistent with the modest genetic diversity and heterozygote deficits described above.

The BayeScan genome scan identified a single outlier locus, Nypa14, which exceeded the FDR = 0.05 decision threshold (posterior probability = 0.9994; log_10_[PO] = 3.22); its comparatively low locus-specific *F*_ST_ (0.049) and negative alpha (α = −1.31) are consistent with balancing selection (**Supplementary Table 7**; **Supplementary Figure 3**). The remaining eleven loci fell below the threshold and were consistent with neutral expectations, supporting their use for population genetic inference.

**Figure 5:**
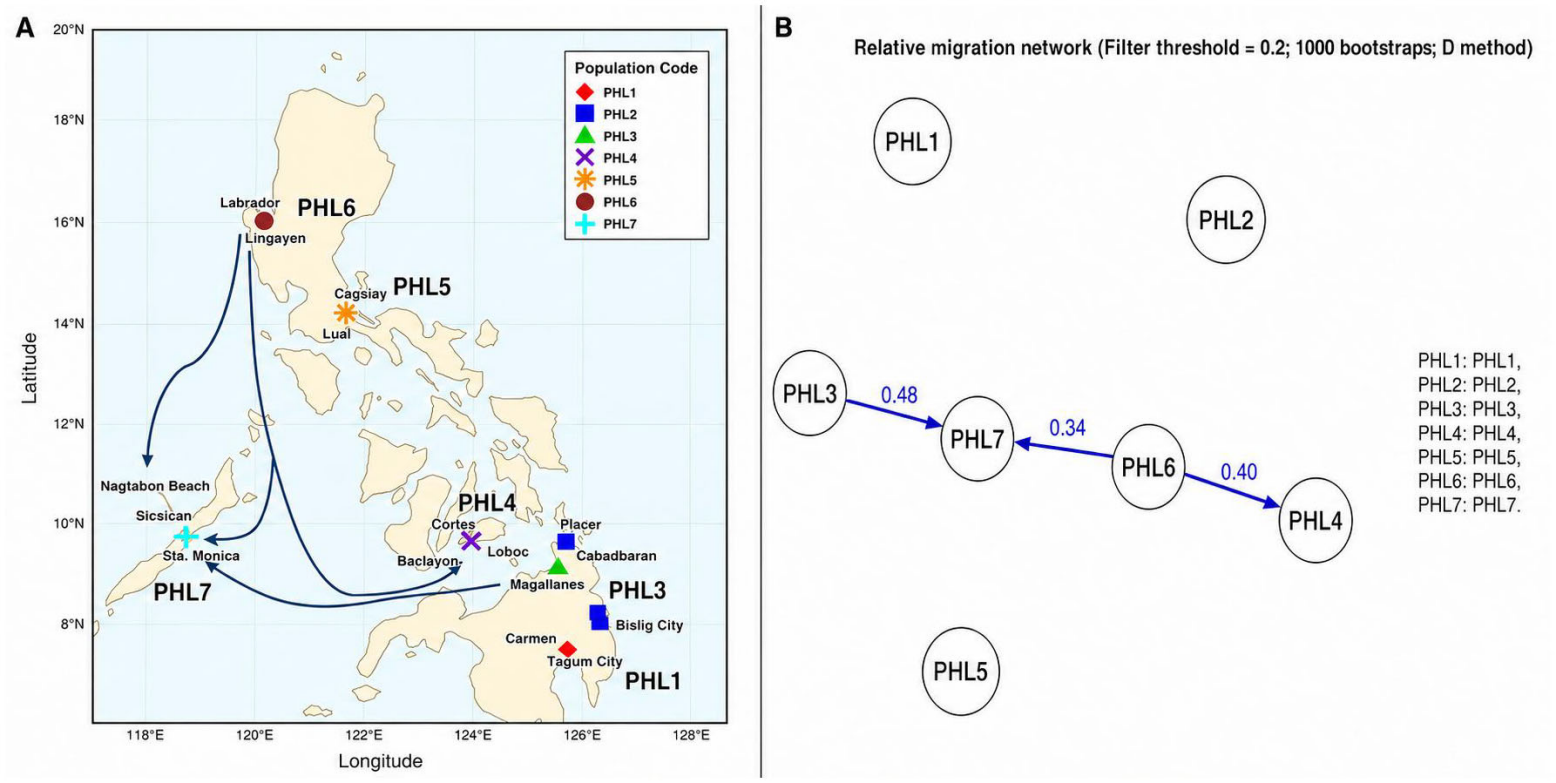
Directional gene flow network among *Nypa fruticans* populations inferred using divMigrate based on Jost’s D. Only statistically supported directional links (1,000 bootstrap replicates, α = 0.05) exceeding a filter threshold of 0.20 are shown. The 0.20 threshold was used as a conservative graphical filter to retain moderate-to-strong directional connections and improve network interpretability. Arrow direction indicates the inferred direction of gene flow, and line thickness is proportional to relative migration strength.

Directional connectivity resulted in a sparse but interpretable migration network (**Figure 5**). After applying a relative migration filter of 0.20 to retain only moderate-to-strong directional links, three population pairs showed statistically supported directional connectivity. The most prominent directional connection was detected from PHL3 to PHL7. In addition, PHL6 showed significant directional links toward both PHL7 and PHL4. No other population pairs displayed statistically supported directional connectivity under these criteria. These results indicate that gene flow across the Philippine archipelago is spatially uneven and concentrated in a limited number of stronger directional pathways. The same three directional pathways were recovered when the network was reconstructed without any filter threshold, confirming that these connections were not an artifact of the visualization cutoff (**Supplementary Figure 2**).

This heterogeneous connectivity pattern parallels the modest but significant population structure detected by AMOVA, PCoA, DAPC, and STRUCTURE. Together, these results indicate that population connectivity across the Philippine archipelago is neither uniform nor negligible, but instead characterized by a limited number of directional connections superimposed on broadly shared genetic variation.

## 4 Discussion

### 4.1 Genetic diversity and demographic context

In this study, genetic profiling of Philippine *N. fruticans* populations across a 12-locus dataset revealed a distinct, semi-connected metapopulation structure. All seven regional populations retained appreciable standing genetic variation, but they were consistently characterized by lower observed than expected heterozygosity, positive *F*_IS_ values, and widespread departures from Hardy–Weinberg equilibrium. The differentiation observed in DAPC should be interpreted with caution because the method utilizes predefined population assignments and is specifically designed to maximize among-group variance. Therefore, DAPC results were evaluated in conjunction with PCoA, STRUCTURE, and AMOVA, which collectively supported the inference of weak but detectable population structure. Collectively, these findings indicate that while these populations share a broadly admixed genetic background, their contemporary structure is distinctly shaped by complex, non-uniform dispersal pathways across the Philippine archipelago.

Philippine populations of *N. fruticans* exhibit a moderate level of genetic diversity. This level falls between the strongly reduced diversity reported for range-edge or bottlenecked mangrove populations and the higher diversity observed in some large, dynamic mangrove systems with substantial standing variation (Triest, 2008; Yahya et al., 2014; Yan et al., 2016). Interestingly, the diversity of the Philippine populations is slightly higher than both the multi-regional Indo-West Pacific average (Mantiquilla et al., 2022) and estimates from several continental Asian localities, including China, Thailand, and Vietnam (Jian et al., 2010). Furthermore, it exceeds the diversity of severely bottlenecked northern-limit populations, such as those on Iriomote Island, Japan, where founder effects and chronic isolation have resulted in markedly reduced allelic richness (Sugai et al., 2016). Overall, this moderate diversity is consistent with values reported for other outcrossing, hydrochorous mangrove species across the Indo-West Pacific, such as *Rhizophora* mangrove species (Wee et al., 2014; Yan et al., 2016).

The genetic diversity observed in Philippine *N. fruticans* occupies an intermediate position relative to other mangrove and *Nypa* populations across the Indo–West Pacific. This intermediate diversity profile suggests that Philippine *N. fruticans* populations have largely avoided extreme genetic erosion. However, they remain vulnerable to demographic fluctuations driven by habitat contraction and localized disturbances. Increasingly, mangrove genetic diversity is understood to be shaped by local geographic heterogeneity rather than simple latitudinal gradients (Triest, 2008; Canty et al., 2022). Our findings strongly align with this perspective, demonstrating that local habitat structure plays a critical role in *N. fruticans*: extensive floodplains and large deltas are capable of sustaining substantial standing genetic variation, whereas populations restricted to smaller or fragmented estuaries are far more prone to episodic diversity loss.

A notable feature of the Philippine dataset is the widespread heterozygote deficit relative to Hardy–Weinberg expectations. Although loci with consistently high null allele frequencies were excluded and remaining loci interpreted conservatively, departures from equilibrium persisted across multiple populations. These residual deficits cannot be attributed solely to genotyping artifacts, but they should not be equated directly with strong biological inbreeding. Empirical and simulation studies demonstrate that null alleles, Wahlund effects, cryptic population subdivision, clonality, and restricted dispersal can all generate positive inbreeding coefficients, often without substantial selfing or close-kin mating (Dakin and Avise, 2004; Chapuis and Estoup, 2007).

The observed heterozygote deficits most likely reflect the combined influence of multiple non-mutually exclusive factors: residual null allele effects at borderline loci (Chapuis and Estoup, 2007), Wahlund effects arising from the pooling of sampling sites with potential fine-scale differentiation (Triest, 2008), and restricted pollen and propagule dispersal generating fine-scale genetic neighborhood structure within estuarine systems. Parentage analyses and spatial genetic studies of other mangrove taxa consistently show that most propagules establish near their maternal origin, producing marked genetic neighbourhoods even within apparently continuous stands (Triest, 2008; Van der Stocken et al., 2019). Thus, *F*_IS_ values in Philippine *N. fruticans* should be viewed primarily as indicators of micro-structuring and departures from panmixia, rather than as evidence of severe or pervasive inbreeding. Positive *F*_IS_ values reflect a statistical excess of homozygotes relative to Hardy–Weinberg expectations, but this departure does not necessarily imply selfing or close-kin mating. In *N. fruticans*, which reproduces primarily by outcrossing through protogynous flowering, high biological inbreeding rates are physiologically unlikely. The elevated *F*_IS_ values reported here are therefore more parsimoniously attributed to restricted propagule dispersal within estuaries, residual null allele effects, and Wahlund effects arising from site pooling (Chapuis and Estoup, 2007; Triest, 2008; Van der Stocken et al., 2019).

### 4.2 Spatial genetic structure within the Philippine archipelago

Across analytical frameworks, Philippine *N. fruticans* populations exhibit weak but statistically significant genetic differentiation. Ordination and clustering approaches (PCoA, DAPC, and STRUCTURE) consistently resolve modest structure characterized by partial separation among regional groups and extensive admixture at the individual level. Such patterns are typical of coastal trees whose dispersal is mediated by ocean currents, where gene flow is sufficient to prevent deep divergence but insufficient to homogenize populations completely (Triest, 2008; Lo et al., 2014; Ngeve et al., 2016).

Within the Philippine archipelago, contrasts among Mindanao, western Luzon, and Palawan relative to Central Visayas and adjacent regions point to the influence of basin-scale geography and circulation. The configuration of major islands and semi-enclosed seas creates heterogeneous dispersal environments, with east–west contrasts imposed by Luzon and its opposing coastal current systems, and north–south discontinuities shaped by the North Equatorial Current and complex inland sea circulation around Mindanao. Comparable patterns of fine-scale differentiation driven by local hydrology and propagule movement have been reported for other Philippine mangroves, including *Avicennia* species (Triest et al., 2021, 2026). Consistent with the broader scale of genetic studies of *N. fruticans* in IWP, which revealed gradients and regional structuring associated with oceanographic connectivity and historical isolation (Mantiquilla et al., 2022), the populations of the Philippines formed part of the mosaic, retaining shared ancestry while exhibiting subtle regional differentiation shaped by the interplay of geography, currents, and dispersal limitation.

### 4.3 Directional gene flow and seascape connectivity

Directional migration analyses further refine this picture by revealing a small number of asymmetric connections among populations. The divMigrate network identifies only a limited set of statistically supported directional links, indicating that gene flow is moderate overall but unevenly distributed across the archipelago. In particular, Palawan (PHL7) receives directional input from both Mindanao (PHL3) and western Luzon (PHL6), while western Luzon also shows connectivity toward Central Visayas (PHL4). Most other population pairs lack robust directional links under conservative filtering criteria. Moreover, the North Equatorial Current of the Pacific Ocean is known to split as it approaches the Philippines with the northern branch known as Kuroshio and the southward bound called Mindanao Current (Qiu et al., 2015). This pattern may become clearer with additional sampling from south-eastern and northern Luzon were sampled since bifurcation is close to these areas. Likewise, if more areas were sampled in eastern, southern and western Mindanao, a clearer connectivity pattern may arise.

This pattern is consistent with the dispersal ecology of mangrove propagules and the hydrodynamic complexity of Philippine seas. According to Han et al. (2009), sea current dynamics in the Sulu Sea are characterized by the convergence of waters from the South China Sea (passing through northern Palawan), the West Pacific (via northern Mindanao), and the Sulawesi Sea, with flow directions dictated by the Asian monsoon. This migration pattern likely explains the connectivity of these populations across the archipelago. Moreover, the strength of connectivity depends on the regularity of the direction of the sea current that resulted in the current population structure. Although *N. fruticans* propagules are buoyant and capable of extended flotation, empirical evidence indicates that successful establishment typically occurs over relatively short distances, with long-distance dispersal events being rare and highly stochastic (Cowen and Sponaugle, 2009; Van der Stocken et al., 2019). Directional connectivity driven by prevailing currents has been documented in multiple mangrove and seagrass systems, producing asymmetric gene flow and localized genetic structure even in species with high dispersal potential (Geng et al., 2021; Nakajima et al., 2023). Together, *N. fruticans* in the Philippines are neither isolated nor fully panmictic, but linked by a small number of dispersal pathways that generate uneven connectivity across regions. Nevertheless, these directional links should be interpreted cautiously because regional sample sizes were unequal, ranging from 12 to 24 individuals. This imbalance may influence allele-frequency-based estimates of relative migration, particularly for smaller populations. Therefore, future studies with larger and more balanced sampling across regions will be needed to confirm the robustness of the inferred directional pathways. The genome scan further indicates that this structure reflects demographic and historical processes rather than selection. With only Nypa14 deviating from neutrality and the remaining loci behaving neutrally, the differentiation recovered across analyses is best attributed to drift and restricted gene flow, reinforcing the validity of these markers for population structure inference.

### 4.4 Historical landscape dynamics and conservation implications

Present-day genetic patterns in Philippine *N. fruticans* likely reflect a strong historical component shaped by Quaternary sea-level fluctuations. During Pleistocene low sea stands, extensive coastal plains, paleo-estuaries, and river systems across the Sunda Shelf would have facilitated widespread connectivity among some coastal populations now separated by deep channels and open seas (Voris, 2000; Sathiamurthy and Voris, 2006). Subsequent Holocene sea-level rise fragmented these systems, leaving remnant estuaries along island margins and river valleys.

Under this scenario, the modest genetic differentiation observed today may represent the residual imprint of a historically more continuous distribution, overlaid by post-glacial fragmentation and contemporary oceanographic forcing. Similar interpretations have been advanced for other coastal taxa, where present-day genetic mosaics arise from the interaction of palaeogeography, dispersal limitation, and ongoing habitat modification (Triest, 2008; Lo et al., 2014).

From a conservation perspective, these findings suggest that the species should be viewed as a semi-connected system of estuarine populations, in which a small number of regions contribute disproportionately to maintaining regional connectivity. Populations in western Luzon and Palawan, which occupy key positions within the inferred dispersal network, may be particularly important for sustaining gene flow and should be prioritised in conservation planning. Maintaining these connectivity routes is especially important because mangrove ecosystems across Southeast Asia are increasingly threatened by land-use conversion, coastal development, hydrological alteration, and other anthropogenic pressures (Richards and Friess, 2016; Friess et al., 2019). Similar conservation challenges have also been highlighted for Indonesian mangrove ecosystems, where biodiversity protection and sustainable management remain major priorities (Suhardi et al., 2024). In addition, future changes in seawater properties may further alter mangrove propagule dispersal potential and modify natural connectivity among estuarine populations (Van der Stocken et al., 2022).

Private allele analysis further substantiates the conservation significance of the proposed key node populations. PHL7 (Palawan) harbored the highest number of private alleles of any population sampled (**Supplementary Table 5**), indicating that it is genetically irreplaceable—loss of this population would permanently remove allelic diversity absent from all other sampled regions. This pattern is mirrored by the distribution of rare alleles (frequency < 0.05), for which PHL7 again recorded the highest count (n = 22; **Supplementary Table 5**). Because rare alleles represent both a reservoir of standing genetic variation and, under drift–migration equilibrium, a signal of gene flow (Slatkin, 1985), their concentration in PHL7 reinforces the conclusion that this population is a disproportionate contributor to the species’ regional gene pool and a priority for safeguarding adaptive potential. PHL6 (western Luzon) carried four private alleles, consistent with its position as a regional connectivity hub. In contrast, PHL1 contained no private alleles, reflecting its peripheral position in the migration network. Effective population size (*N*_e_) were uniformly small across all populations (*N*_e_  = 12.1–32.3; **Supplementary Table 6**), with PHL3 recording the lowest estimate (*N*_e_  = 12.1; 95% CI: 5.8–34.3) - below the *Ne* = 50 threshold for short-term demographic viability (Franklin, 1980; Frankham et al., 2014). The wide parametric confidence intervals reflect the inherent limitation of linkage disequilibrium–based Ne estimation with small sample sizes and should be interpreted as indicative. Collectively, these findings provide quantitative genetic support for prioritizing PHL7, PHL6, and PHL3 in conservation planning for Philippine *Nypa fruticans.*

An additional limitation of this study is that several sampling sites were combined into broader regional units to represent shared estuarine and coastal systems. Although this approach was defined prior to genetic analyses and reflects expected dispersal settings, finer-scale subdivision may remain undetected within some regions. Future studies incorporating denser spatial sampling will help evaluate potential within-region structure and refine connectivity estimates.

Finally, this analysis provides an empirical Philippine baseline for future regional comparisons with *N. fruticans* populations across Southeast Asia, including Malaysia and Thailand. By integrating carefully filtered microsatellite data with an explicit seascape and paleogeographic framework, this study highlights *N. fruticans* as a valuable model for understanding how estuarine specialists respond genetically to the combined influences of coastal fragmentation, ocean circulation, and long-term sea-level change in Southeast Asia.

## 5 Conclusion

Taken together, Philippine *Nypa fruticans* populations retain moderate genetic diversity across estuarine habitats, without evidence of extreme genetic erosion at a national scale. At the same time, genetic variation is unevenly distributed among regions, reflecting differences in estuarine configuration and demographic history. Across analytical approaches, populations exhibit weak but detectable spatial differentiation superimposed on a broadly shared genetic background. Ordination and clustering analyses reveal partial regional structuring, while directional migration analyses indicate that connectivity is asymmetric and concentrated along a limited number of dispersal pathways. These results collectively support a semi-connected population system in which local demographic processes generate subtle differentiation, yet regional gene flow prevents deep fragmentation.

By integrating diversity, structure, and directional connectivity within an explicitly archipelagic framework, this study provides a coherent view of how an estuarine specialist responds to heterogeneous seascapes. The Philippine case establishes a genetic baseline for broader regional comparisons and underscores the importance of maintaining connectivity among key estuarine systems in conservation planning. These findings have important implications for conservation planning in the Philippine archipelago. In particular, Palawan (PHL7) is genetically irreplaceable, harboring the highest numbers of private and rare alleles, while western Luzon (PHL6) occupies a key node within the inferred connectivity network that sustains regional gene flow, and the Agusan Basin population (PHL3), although not a connectivity hub, shows the lowest effective population size and is therefore the most demographically vulnerable. Conserving these regions—prioritized on the complementary grounds of genetic distinctiveness, connectivity, and demographic vulnerability—together with the dispersal pathways linking them to other estuarine systems should therefore be considered a priority in future mangrove management and restoration programs.

## Data Availability

The original contributions presented in this study are included in the article and **Supplementary Material**. Further inquiries can be directed to the corresponding authors.
