# Supplementary material for: Semi-connected structure and asymmetric gene flow of Nypa fruticans Wurmb. across the Philippine archipelago
Source: Front Plant Sci. 2026 Jul 6;17:1879288. doi: 10.3389/fpls.2026.1879288 (PMC13381821; doi:10.3389/fpls.2026.1879288)
Supplement: Supplementary file 1 [file DataSheet1.pdf]

Supplementary Materials

Supplementary Table S1. Primer sequences, annealing temperatures (Tm), and expected amplicon size ranges for the 18 microsatellite loci used for initial genotyping of *N. fruticans*. These loci were screened prior to null-allele filtering, and the final population genetic analyses were conducted using the retained 12-locus dataset.

**Primer code**	**Sequences**		**Tm (°C)**	**Amplicon size (bp)**
Nypa1	F:	CCATCAGGCCAAACAGTTCAG	54	276 – 296
	R:	TCTTGCCGGTCCGTTTAAGA		
Nypa2	F:	GGATTTTGGCCGTGGCTTTT	58	258 – 310
	R:	ACATTTCTCGGACGGGTTGG		
Nypa3	F:	ATGAGAACCCATGCCGATGT	58	294 – 314
	R:	TGATCGACTTATAGTCAGCCCT		
Nypa4	F:	GGTAACGTCCTCTCCAACCA	59	202 – 210
	R:	TCCATGGCAGGAAGTTTGCT		
Nypa5	F:	AGTGAGAGATGGGGATTTGTTGA	58	262 – 298
	R:	GCAAGATATAGGTTGCCGGTC		
Nypa6	F:	CGCTTGTTCTTTTGGCTTGC	58	224 – 298
	R:	AGCCATGGCCATGATGATCA		
Nypa7	F:	TGGCACAACCATCCAACAAC	59	264 – 304
	R:	AGATATTGTGGGGCCCAAACA		
Nypa8	F:	TGCCGTCCTGATCATCGTTT	55	216 – 250
	R:	TGGTGCACATGGATCTCAGT		
Nypa9	F:	ACAATGCCTCCAACAGCTCA	59	100 – 106
	R:	AAAAAGCTTGCACCCACACA		
Nypa10	F:	GCCCACACTCTGTTACCCAA	59	258 – 290
	R:	TGCATAATCAGCTTGCGTGA		
Nypa11	F:	ACACACACATACACACACACAG	52	156 – 168
	R:	TTACCGGACCCACGTTGAAG		
Nypa12	F:	GCTTGCTGCTGCTGTACTTC	52	174 – 194
	R:	TGGATGTTTTGGTGTGGTTGG		
Nypa13	F:	GGGAGCTCTCGCATATCTCC	58	154 – 182
	R:	TCCTCCTTTGCATGGTGGTC		
Nypa14	F:	GTCGGCGAGCTAGGGTTTAA	59	280 – 300
	R:	AGCCATGAATATTCCCCTCAAA		
Nypa15	F:	GTCATTGTTCGCACCGTCAG	59	160 – 182

	R:	AGGGTGGTTGTTGCGATGAT		
Nypa16	F:	GCACTTGCAAGCTCCATCAG	58	228 – 246
	R:	GGCCGGTCAGGAGTTAAACA		
Nypa17	F:	TGCACAGGTCAAACGTAGGA	54	260 – 280
	R:	TGCATTTGGAGCCCATTTTTCT		
Nypa18	F:	TTGCTTGGGGCCAACAATTG	52	178 – 190
	R:	TCGCAAACTTAAGGACCTGGA		

Supplementary Table S2. Estimated mean null allele frequencies for 18 microsatellite loci across sampled Philippine populations of *Nypa fruticans*. High frequencies (>0.20) may indicate the presence of null alleles which can contribute to the observed heterozygote deficits

**Locus**	**Null_Allele_Frequency**
Nypa1	0.407
Nypa2	0.474
Nypa3	0.335
Nypa4†	0.903
Nypa5	0.419
Nypa6†	0.636
Nypa7	0.404
Nypa8	0.465
Nypa9	0.129
Nypa10	0.468
Nypa11†	0.817
Nypa12†	0.662
Nypa13†	0.508
Nypa14	-0.002
Nypa15	0.321
Nypa16	0.492
Nypa17	0.315
Nypa18†	1.000

Note: †Loci excluded from downstream analyses because they showed consistently high null-allele frequencies across populations.

Supplementary Table S3. Summary of Chi-Square tests for departures from Hardy-Weinberg Equilibrium (HWE) across regional Philippine populations of *Nypa fruticans*. Significant deviations from HWE are indicated by asterisks. Key: ns = not significant; * P < 0.05; ** P < 0.01; *** P < 0.001

Pop	Locus	DF	ChiSq	Prob	Signif
PHL1	Nypa1	10	39.648	0.000	***
PHL1	Nypa2	3	15.150	0.002	**
PHL1	Nypa3	3	15.034	0.002	**
PHL1	Nypa5	15	34.169	0.003	**
PHL1	Nypa7	3	30.000	0.000	***
PHL1	Nypa8	15	75.000	0.000	***
PHL1	Nypa9	1	0.000	0.985	ns
PHL1	Nypa10	15	27.308	0.026	*
PHL1	Nypa14	28	31.200	0.308	ns
PHL1	Nypa15	6	9.417	0.151	ns
PHL1	Nypa16	6	17.140	0.009	**
PHL1	Nypa17	21	47.333	0.001	***
PHL2	Nypa1	15	48.401	0.000	***
PHL2	Nypa2	10	51.122	0.000	***
PHL2	Nypa3	21	81.234	0.000	***
PHL2	Nypa5	15	37.518	0.001	**
PHL2	Nypa7	10	73.140	0.000	***
PHL2	Nypa8	10	27.278	0.002	**
PHL2	Nypa9	6	21.183	0.002	**
PHL2	Nypa10	15	46.325	0.000	***
PHL2	Nypa14	21	52.463	0.000	***
PHL2	Nypa15	6	19.519	0.003	**
PHL2	Nypa16	28	93.389	0.000	***
PHL2	Nypa17	36	49.398	0.068	ns
PHL3	Nypa1	6	13.775	0.032	*
PHL3	Nypa2	1	1.333	0.248	ns
PHL3	Nypa3	10	36.374	0.000	***
PHL3	Nypa5	10	31.866	0.000	***
PHL3	Nypa7	3	12.344	0.006	**
PHL3	Nypa8	6	24.480	0.000	***
PHL3	Nypa9	1	0.310	0.577	ns
PHL3	Nypa10	3	24.000	0.000	***
PHL3	Nypa14	15	21.000	0.137	ns
PHL3	Nypa15	6	14.265	0.027	*
PHL3	Nypa16	15	24.013	0.065	ns
PHL3	Nypa17	21	53.653	0.000	***
PHL4	Nypa1	15	75.052	0.000	***
PHL4	Nypa2	15	33.459	0.004	**
PHL4	Nypa3	15	61.465	0.000	***
PHL4	Nypa5	10	45.917	0.000	***
PHL4	Nypa7	15	54.875	0.000	***
PHL4	Nypa8	28	92.728	0.000	***
PHL4	Nypa9	1	2.798	0.094	ns
PHL4	Nypa10	6	47.058	0.000	***
PHL4	Nypa14	15	57.214	0.000	***
PHL4	Nypa15	15	54.234	0.000	***
PHL4	Nypa16	21	66.600	0.000	***
PHL4	Nypa17	15	39.185	0.001	***
PHL5	Nypa1	6	20.026	0.003	**
PHL5	Nypa2	15	65.400	0.000	***
PHL5	Nypa3	10	10.708	0.381	ns
PHL5	Nypa5	15	10.474	0.789	ns
PHL5	Nypa7	10	37.861	0.000	***
PHL5	Nypa8	28	49.819	0.007	**
PHL5	Nypa9	1	4.863	0.027	*
PHL5	Nypa10	6	30.124	0.000	***
PHL5	Nypa14	36	90.000	0.000	***
PHL5	Nypa15	3	15.052	0.002	**
PHL5	Nypa16	10	20.841	0.022	*
PHL5	Nypa17	15	48.000	0.000	***
PHL6	Nypa1	1	18.000	0.000	***
PHL6	Nypa2	10	51.844	0.000	***
PHL6	Nypa3	1	18.000	0.000	***
PHL6	Nypa5	6	31.874	0.000	***
PHL6	Nypa7	6	33.034	0.000	***
PHL6	Nypa8	1	0.720	0.396	ns
PHL6	Nypa9	1	0.720	0.396	ns
PHL6	Nypa10	15	43.103	0.000	***
PHL6	Nypa14	21	33.535	0.041	*
PHL6	Nypa15	6	12.182	0.058	ns
PHL6	Nypa16	21	90.519	0.000	***
PHL6	Nypa17	15	33.660	0.004	**
PHL7	Nypa1	10	23.620	0.009	**
PHL7	Nypa2	36	76.363	0.000	***
PHL7	Nypa3	21	33.047	0.046	*
PHL7	Nypa5	36	59.483	0.008	**
PHL7	Nypa7	6	14.011	0.030	*
PHL7	Nypa8	21	63.750	0.000	***
PHL7	Nypa9	1	1.195	0.274	ns
PHL7	Nypa10	15	28.871	0.017	*
PHL7	Nypa14	15	52.114	0.000	***
PHL7	Nypa15	6	11.730	0.068	ns
PHL7	Nypa16	21	36.667	0.018	*
PHL7	Nypa17	21	60.600	0.000	***

Supplementary Table S4. Evanno table output for determining the optimal number of genetic clusters (K) in *Nypa fruticans.*

K	Reps	Mean LnP(K)	Stdev LnP(K)	Ln'(K)	|Ln''(K)|	Delta K
1	10	-4776.13000	0.33682	NA	NA	NA
**2**	**10**	**-4532.88000**	**0.46857**	**243.25000**	**85.89000**	**183.30334**
3	10	-4375.52000	12.77913	157.36000	12.08000	0.94529
4	10	-4230.24000	1.01675	145.28000	23.80000	23.40795
5	10	-4108.76000	1.31335	121.48000	54.32000	41.35987
6	10	-4041.60000	2.49978	67.16000	9.10000	3.64032
7	10	-3965.34000	6.15453	76.26000	4.95000	0.80429
8	10	-3894.03000	1.65667	71.31000	32.30000	19.49694
9	10	-3855.02000	4.96763	39.01000	20.42000	4.11061
10	10	-3795.59000	3.11999	59.43000	NA	NA

Supplementary Table S5. Number of private alleles and rare alleles per population for *Nypa fruticans* sampled across the Philippines (12 microsatellite loci, n = 118)

**Population**	**n**	**N_Private_Alleles**	**N_Rare_Alleles**
PHL1	15	0	8
PHL2	24	6	20
PHL3	12	1	5
PHL4	19	7	15
PHL5	15	7	15
PHL6	18	4	8
**PHL7**	**15**	**11**	**22**
Total	118	36	93

Supplementary Table S6. Effective population size (N_e) estimates for seven *Nypa fruticans* populations across the Philippines, generated by NeEstimator v2.1 (Do et al., 2014).

Population	Sample Size (N)	N_e (LD)	95% CI Parametric	95% CI Jackknife	N_e (Molecular Coancestry)
PHL1	15	21.7	11.2– 71.7	7.1–∞	6.8
PHL2	24	32.3	20.0–66.5	16.6–115.6	4.7
PHL3	12	12.1	5.8–34.3	2.7–∞	8.5
PHL4	19	27.6	16.8–58.4	12.9–156.1	73.6
PHL5	15	24.9	13.4–75.3	7.3–∞	6.2
PHL6	18	23.0	12.0–69.5	7.7–∞	20.4
PHL7	15	28.2	15.9–76.4	11.4–∞	7.0

* N_e = effective population size estimated using the LD method (Hill, 1981; Waples & Do, 2008). ∞ upper CI bounds indicate insufficient data to constrain the upper limit, expected with small sample sizes (n = 12–24). All N_e *estimated using the LD method* < 50 (Frankham, 2014 vulnerability threshold). Yellow = PHL3 (lowest N_e); green = key node populations PHL6 and PHL7. Heterozygote excess method returned ∞ for all populations (no acute bottleneck signal).

Supplementary Table S7. BayeScan analysis of selection in microsatellite loci of *Nypa fruticans* populations in the Philippines. For each locus, the posterior probability (Prob) of being under selection, the base-10 logarithm of the posterior odds ($\log_{10}$(PO)), the locus-specific alpha (α), and the locus-specific F_{ST} are reported. A positive α indicates diversifying selection, whereas a negative α indicates balancing (or purifying) selection.

Locus	Prob	$\log_{10}$(PO)	α (alpha)	F_{ST}
Nypa1	0.0574	-1.2153	-0.0208	0.1462
Nypa2	0.0402	-1.3779	0.0094	0.1496
Nypa3	0.0354	-1.4353	-0.0053	0.1478
Nypa5	0.0322	-1.4778	-0.0039	0.1479
Nypa7	0.0700	-1.1233	0.0293	0.1525
Nypa8	0.0296	-1.5156	0.0035	0.1488
Nypa9	0.0564	-1.2234	0.0108	0.1501
Nypa10	0.0418	-1.3602	-0.0034	0.1481
Nypa14	0.9994	3.2215	-1.3134	0.0487
Nypa15	0.0506	-1.2732	-0.0152	0.1468
Nypa16	0.8332	0.6985	-0.7164	0.0841
Nypa17	0.3455	-0.2775	-0.2293	0.1253

*Only Nypa14 exceeded the FDR = 0.05 decision threshold ($\log_{10}$(PO) = 3.22; α = −1.31; F_{ST} = 0.049); its low locus-specific F_{ST} and negative α are consistent with balancing selection, which maintains allelic diversity across populations and does not bias directional gene flow estimates. The remaining eleven loci fell below the threshold and are interpreted as evolving neutrally.

Prob = posterior probability of the model including selection; $\log_{10}$(PO) = base-10 logarithm of the posterior odds; α = locus-specific alpha (locus effect indicating the strength and direction of selection); F_{ST} = locus-specific F_{ST} averaged over populations. Analysis performed using BayeScan v2.01 (Foll and Gaggiotti, 2008); 20 pilot runs × 5,000 iterations; burn-in = 50,000; thinning = 10; total MCMC = 100,000.

Supplementary Figure S1. *K*-selection statistics from STRUCTURE analysis of *Nypa fruticans* (Philippines, 12 SSR loci, n = 118 individuals, 10 runs per K).

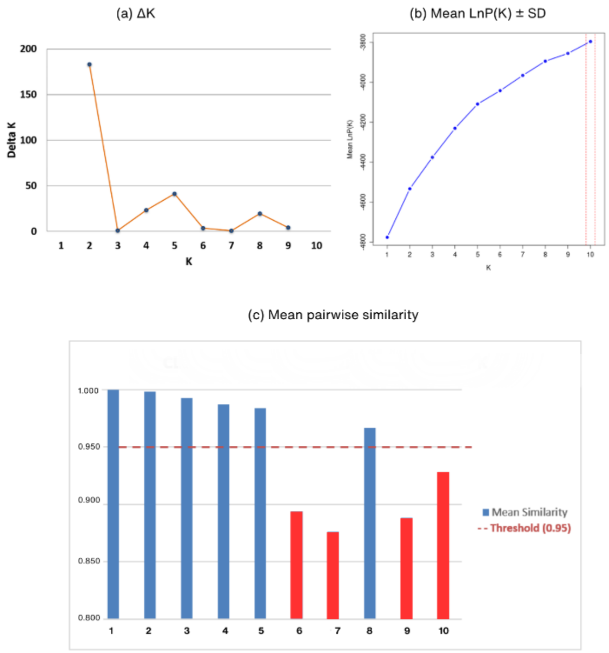

(a) A dominant peak at $K = 2$ ($\Delta K = 183.3$) indicates that two ancestry clusters receive the strongest statistical support. A secondary peak at $K = 5$ ($\Delta K = 41.4$) was observed but not further interpreted, as the corresponding partition was not concordant with DAPC or other complementary clustering analyses. (b) Mean LnP(K) ± SD across 10 replicate runs per K value. The curve increases steeply from $K = 1$ to $K = 2$ and then plateaus, consistent with $K = 2$ as the primary clustering solution identified by ΔK. (c) Mean pairwise similarity among replicate runs, computed by CLUMPP (Jakobsson & Rosenberg, 2007) within CLUMPAK (Kopelman et al., 2015). Blue bars indicate K values where mean similarity ≥ 0.95 (replicate runs converged to the same solution); red bars indicate K values where similarity < 0.95 (runs were unstable). Together, panels (a)–(c) consistently support $K = 2$ as the optimal number of genetic clusters.

Supplementary Figure S2. Complete directional gene flow network inferred by divMigrate without application of a visualization threshold (filter threshold = 0; 1,000 bootstrap replicates; Jost's D method). All statistically supported migration pathways are displayed.

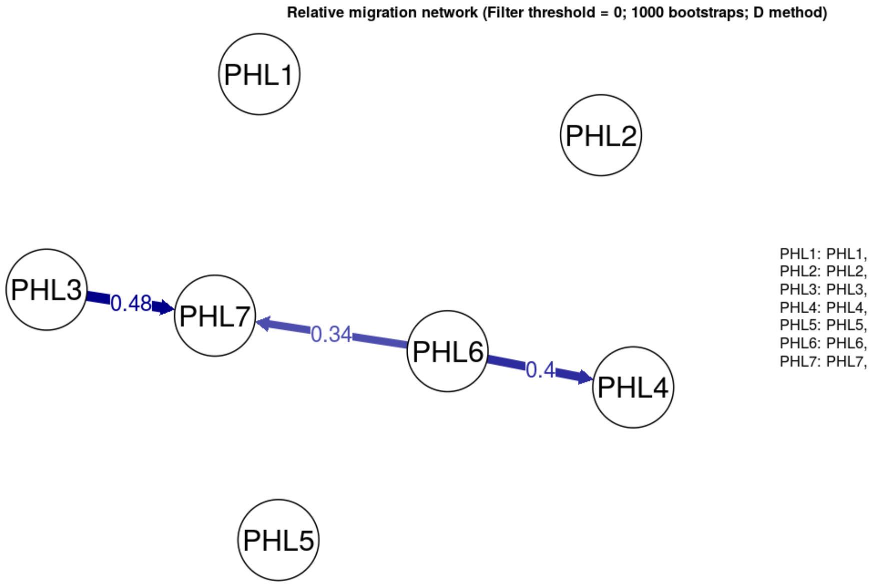

Supplementary Figure S3. BayeScan genome-scan plot identifying candidate loci under selection in *Nypa fruticans* sampled across the Philippines.

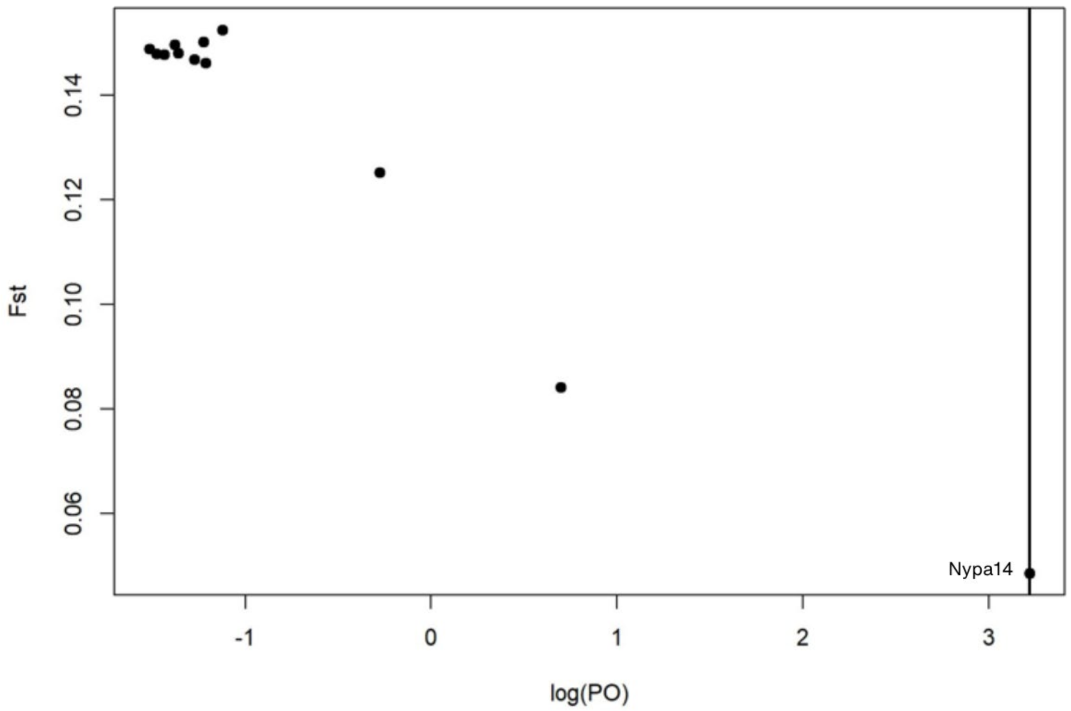

*Each point represents one of the 12 microsatellite loci, plotted as locus-specific F_{ST} against $\log_{10}$ of the posterior odds ($\log_{10}$[PO]). The vertical line marks the FDR = 0.05 threshold. One locus, Nypa14, falls beyond the threshold as an outlier; its low F_{ST} (0.0487) and negative α (−1.3134) are consistent with balancing selection. The remaining eleven loci are interpreted as neutral.

Supplementary Raw Data Sheet 1. Raw microsatellite genotype matrix of *Nypa fruticans* individuals from the Philippine archipelago. The sheet contains the individual sample code, regional population assignment, and paired allele-size data for the 12 microsatellite loci retained after null-allele filtering. These genotypes constitute the final dataset used for downstream population genetic analyses.

12	118	7	15	24	12	19	15	18	15	1	118																
			PHL1	PHL2	PHL3	PHL4	PHL5	PHL6	PHL7																		
code	pop	Nypa1		Nypa2		Nypa3		Nypa5		Nypa7		Nypa8		Nypa9		Nypa10		Nypa14		Nypa15		Nypa16		Nypa17			
CD05	PHL1	292	292	268	266	300	300	268	268	292	292	234	234	104	104	276	270	294	284	162	162	234	234	264	264		
CD07	PHL1	292	292	268	266	300	300	266	266	292	292	234	234	104	100	276	270	286	286	162	162	236	234	264	264		
CD19	PHL1	292	286	270	270	312	300	268	268	292	292	238	238	100	100	280	272	294	282	180	162	238	238	270	270		
TD04	PHL1	288	288	268	268	312	312	266	266	292	292	230	230	104	100	276	276	296	284	180	164	236	236	274	266		
TD11	PHL1	292	288	268	268	312	300	284	264	292	292	224	224	104	100	278	274	284	284	178	162	238	238	264	264		
TD15	PHL1	292	292	268	268	312	300	284	264	290	290	224	224	104	104	278	278	290	284	178	162	238	236	272	264		
TD16	PHL1	292	292	268	268	312	300	264	264	292	292	226	226	104	100	278	274	292	284	178	162	238	238	264	264		
TD20	PHL1	292	292	268	268	300	300	278	268	290	290	226	226	104	104	278	278	292	284	162	162	238	238	264	264		
TD21	PHL1	292	292	268	268	312	300	278	264	292	292	224	224	104	100	278	278	292	284	180	180	236	236	264	264		
TD22	PHL1	284	284	270	270	300	300	268	268	294	294	232	232	104	100	278	270	286	286	164	164	236	236	266	266		
TD26	PHL1	290	290	268	268	300	300	284	268	292	292	230	230	100	100	278	278	288	284	178	178	240	236	268	268		
TD27	PHL1	292	292	270	270	300	300	268	268	294	294	234	234	104	104	272	272	294	282	162	162	238	238	268	268		
TD30	PHL1	290	290	270	270	300	300	298	284	292	292	234	234	104	100	278	278	294	284	180	162	240	236	270	270		
TD31	PHL1	288	288	268	268	300	300	298	284	294	294	230	230	104	104	274	274	294	286	180	164	238	238	264	264		
TD33	PHL1	288	288	268	268	310	310	264	264	292	292	234	234	104	104	280	274	294	282	162	162	236	236	278	268		
BS03	PHL2	292	288	268	268	312	312	266	266	292	292	230	230	104	104	276	276	292	284	162	162	234	234	272	272		
BS04	PHL2	290	290	266	266	312	312	262	262	292	292	230	230	104	100	276	276	294	284	162	162	236	236	272	264		
BS05	PHL2	288	288	266	266	300	300	0	0	294	294	230	230	106	106	278	278	290	284	162	162	240	240	274	268		
BS06	PHL2	292	292	270	270	312	300	268	268	292	282	232	232	104	100	276	258	290	284	178	164	236	236	274	266		
BS07	PHL2	290	286	268	268	312	312	266	266	298	298	230	230	104	104	276	276	292	284	162	162	234	234	264	264		
BS08	PHL2	288	288	268	268	312	300	268	268	294	294	230	230	104	104	278	278	294	282	162	162	238	238	280	266		
BS09	PHL2	294	294	0	0	302	302	268	268	292	292	236	236	104	102	276	258	284	284	164	164	0	0	274	266		
BS10	PHL2	0	0	0	0	310	300	268	268	292	292	232	232	104	102	276	260	294	294	164	164	236	232	272	266		
BS11	PHL2	292	292	268	268	310	302	268	268	292	282	236	230	104	104	276	258	294	282	164	164	236	236	272	266		
BS15	PHL2	288	288	268	268	310	310	266	266	292	282	236	230	106	106	274	274	294	282	162	162	238	238	278	278		
BS16	PHL2	284	284	0	0	312	302	268	268	292	282	238	232	106	106	278	278	284	284	164	164	240	240	278	270		
BS19	PHL2	288	284	266	266	310	310	268	264	292	282	238	232	104	104	278	278	294	282	162	162	238	238	274	266		
BS20	PHL2	292	288	270	270	312	312	0	0	292	282	236	230	104	104	274	274	294	282	162	162	240	240	276	268		
BS21	PHL2	288	288	268	268	312	312	266	266	292	282	236	230	104	104	278	278	294	284	162	162	240	240	276	276		
BS22	PHL2	290	290	272	272	310	300	268	266	282	282	0	0	106	106	276	276	284	284	164	164	240	240	278	268		
BS23	PHL2	284	284	266	266	312	300	266	266	292	292	238	232	104	104	278	278	290	284	162	162	238	238	274	266		
BS24	PHL2	294	290	0	0	306	306	286	268	298	298	236	236	106	102	278	258	290	284	180	164	236	230	268	268		
BS26	PHL2	294	294	266	266	314	302	268	268	292	282	240	236	106	106	278	278	286	286	164	164	242	242	274	266		
PS09	PHL2	288	284	268	268	294	294	278	266	290	290	236	236	104	100	276	272	294	282	164	164	236	236	266	266		
PS18	PHL2	288	288	268	266	294	294	278	268	292	292	236	236	104	104	276	276	294	284	180	164	236	236	266	266		
PS23	PHL2	288	288	268	266	310	310	266	266	292	292	236	236	104	104	276	272	288	282	180	164	236	236	266	266		
PS25	PHL2	288	284	268	268	314	302	268	268	292	282	236	230	104	104	278	278	294	284	180	162	240	240	276	266		
PS28	PHL2	288	288	268	264	314	302	278	266	292	282	236	236	104	100	276	276	294	284	162	162	240	240	266	266		
PS33	PHL2	292	292	268	268	300	300	268	268	292	292	238	238	104	104	276	276	294	282	178	162	234	228	272	264		
CA04	PHL3	292	288	268	268	312	300	266	266	292	292	236	236	104	100	278	278	294	286	162	162	238	238	264	264		
CA17	PHL3	288	284	268	266	314	302	266	266	290	290	236	236	104	100	278	278	294	284	180	176	240	240	276	276		
CA18	PHL3	288	288	268	266	312	312	284	268	292	292	230	226	104	100	274	274	294	284	162	162	236	236	278	270		
CA20	PHL3	290	290	268	268	312	312	266	266	292	282	230	226	104	100	276	276	294	282	180	180	238	230	278	270		
CA23	PHL3	288	284	268	266	312	312	278	278	292	292	236	236	104	100	278	278	292	286	180	164	240	234	276	276		
CA28	PHL3	288	284	268	268	312	300	268	266	282	282	236	236	100	100	278	278	288	284	164	164	238	234	268	268		
MA06	PHL3	288	288	268	266	310	310	268	268	292	282	230	230	104	104	278	278	292	282	180	164	234	234	274	266		
MA08	PHL3	288	284	268	268	312	300	268	268	292	282	230	230	104	104	278	278	292	282	164	164	238	238	276	268		
MA16	PHL3	288	288	268	266	312	312	266	266	290	290	230	230	104	104	276	276	294	282	180	164	240	232	278	270		
MA18	PHL3	288	288	268	266	312	312	264	264	290	290	230	230	100	100	276	276	294	284	162	162	240	240	266	266		
MA19	PHL3	290	290	268	268	312	312	266	266	292	292	228	228	100	100	276	276	294	284	162	162	238	238	268	268		
MA24	PHL3	290	290	268	268	312	312	266	266	292	292	228	228	100	100	276	276	294	284	180	164	238	238	278	270		
BB07	PHL4	292	292	268	268	312	300	262	262	292	282	228	224	104	100	278	278	292	284	162	162	234	234	264	264		
BB11	PHL4	292	292	268	268	300	300	264	264	282	282	228	228	104	104	276	276	290	284	162	162	244	234	264	264		
BB16	PHL4	288	288	300	270	306	306	266	266	304	286	238	218	104	104	280	280	290	290	166	160	234	234	274	266		
CB04	PHL4	288	284	266	266	300	300	278	266	292	292	232	232	104	100	278	278	290	284	180	180	240	240	276	268		
CB05	PHL4	294	290	268	268	310	300	268	268	282	282	234	234	100	100	276	276	290	284	164	164	228	228	274	264		
CB06	PHL4	282	282	268	268	312	302	268	268	292	292	234	234	104	100	280	276	290	290	178	178	232	232	278	268		
CB10	PHL4	294	290	266	266	300	300	268	268	292	282	236	236	104	100	276	276	288	288	180	164	234	234	264	264		
CB19	PHL4	288	284	268	264	300	300	264	264	292	292	232	232	104	100	278	278	290	284	164	164	240	240	276	268		
CB33	PHL4	288	284	268	266	294	294	264	264	290	290	230	230	100	100	276	276	288	288	180	164	232	232	266	266		
CB34	PHL4	0	0	270	268	300	300	268	268	292	292	224	224	104	100	280	280	292	292	180	164	238	238	268	268		
CB38	PHL4	282	282	268	268	300	300	268	268	292	282	234	234	104	100	280	276	294	282	178	162	238	238	268	268		
CB39	PHL4	288	284	264	264	310	310	268	268	288	288	234	224	104	100	276	276	288	288	180	162	238	238	274	266		
CB43	PHL4	288	284	268	268	300	300	268	266	290	290	232	232	104	100	278	278	288	288	164	164	240	234	276	268		
LB02	PHL4	288	288	268	264	300	300	264	264	292	282	236	230	104	100	280	280	288	284	164	164	236	232	266	266		
LB03	PHL4	288	288	270	270	312	312	266	266	304	290	230	230	104	104	280	280	288	284	180	164	240	232	274	266		
LB07	PHL4	288	284	264	260	312	302	266	266	290	290	234	234	104	100	276	276	288	284	180	162	236	236	274	266		
LB10	PHL4	288	288	268	268	312	300	266	264	292	282	236	236	104	100	280	280	294	282	180	164	238	238	264	264		
LB16	PHL4	288	284	264	264	310	310	266	266	292	282	236	230	104	100	274	274	294	282	162	162	238	238	266	266		
LB25	PHL4	288	288	264	264	300	300	266	266	292	282	230	230	104	104	274	274	294	282	180	162	236	236	266	266		
CQ03	PHL5	288	288	264	264	312	300	284	268	304	304	230	224	104	100	280	280	294	282	162	162	240	240	274	274		
CQ04	PHL5	292	288	290	264	300	294	268	262	304	292	240	220	104	104	276	270	296	284	162	162	238	234	272	264		
CQ05	PHL5	288	288	290	290	312	302	266	266	302	302	230	230	100	100	278	278	296	284	164	164	232	232	272	272		
CQ06	PHL5	292	292	288	288	300	300	284	284	300	300	220	220	104	104	278	278	298	286	162	162	238	234	272	272		
CQ11	PHL5	288	284	288	288	312	306	266	266	304	304	242	224	104	104	280	280	294	282	164	164	234	234	274	266		
CQ14	PHL5	288	284	264	264	306	300	268	264	300	292	242	222	104	100	276	276	300	286	164	164	240	234	272	272		
CQ16	PHL5	292	288	290	290	312	306	268	266	302	302	240	220	104	104	278	278	294	282	162	162	234	234	272	264		
LQ01	PHL5	288	284	290	264	306	294	284	268	300	300	224	224	104	104	280	280	294	282	162	162	234	234	276	276		
LQ05	PHL5	288	288	270	268	302	302	268	262	302	302	222	222	104	104	278	278	292	286	164	164	236	232	272	272		
LQ11	PHL5	292	292	290	290	300	300	268	268	298	298	240	240	100	100	276	276	294	282	162	162	238	238	274	274		
LQ12	PHL5	288	288	266	266	306	300	284	268	300	300	242	222	104	100	276	276	292	286	164	164	236	232	274	266		
LQ14	PHL5	292	292	264	264	312	300	268	268	300	300	224	224	104	104	278	278	294	282	178	162	238	232	274	274		
LQ18	PHL5	290	290	290	290	306	300	286	266	298	298	236	236	100	100	278	278	294	282	162	162	234	234	278	278		
LQ20	PHL5	288	288	266	266	306	300	284	266	304	304	230	230	100	100	276	276	292	286	164	164	236	232	274	266		
LQ21	PHL5	292	288	264	264	300	300	268	268	302	292	234	224	104	104	276	276	290	290	162	162	232	232	274	266		
LP02	PHL6	284	284	270	270	300	300	284	284	290	290	236	236	104	104	278	278	292	284	178	178	234	234	276	268		
LP04	PHL6	284	284	270	270	300	300	284	266	290	290	236	236	104	104	280	272	292	284	180	180	232	232	276	268		
LP09	PHL6	284	284	268	268	300	300	284	266	290	290	236	230	104	100	278	278	294	280	180	164	234	230	268	268		
LP12	PHL6	284	284	290	268	300	300	278	278	304	292	236	230	104	100	278	278	294	280	180	164	236	236	266	266		
LP21	PHL6	284	284	290	268	300	300	278	266	292	292	236	236	104	104	284	280	290	284	180	180	236	236	266	266		
LP25	PHL6	284	284	268	266	300	300	278	278	304	292	236	230	104	100	278	278	292	292	180	180	234	234	276	266		
PP03	PHL6	284	284	290	268	300	300	276	276	304	292	236	236	104	104	284	280	294	284	180	180	238	238	276	266		
PP11	PHL6	284	284	290	268	300	300	284	284	304	292	236	236	104	104	278	278	294	282	178	178	246	240	278	268		
PP12	PHL6	284	284	290	268	302	302	276	276	304	292	236	236	104	104	282	278	294	284	180	180	238	238	276	266		
PP16	PHL6	284	284	268	268	300	300	284	266	290	290	236	236	104	104	284	280	294	282	178	178	234	234	268	268		
PP23	PHL6	284	284	268	268	300	300	284	284	304	290	236	236	104	104	278	278	292	284	178	164	232	232	266	266		
PP29	PHL6	284	284	266	266	300	300	284	284	302	302	236	230	104	100	280	276	288	284	180	162	234	234	266	266		
PP33	PHL6	288	288	268	268	302	302	284	266	304	304	236	230	104	100	278	278	294	284	180	164	230	230	264	264		
PP37	PHL6	288	288	266	266	302	302	266	266	292	292	236	236	104	104	278	278	294	282	180	180	230	230	264	264		
PP38	PHL6	284	284	266	266	302	302	278	278	304	292	236	236	104	104	276	276	288	284	178	178	234	234	266	266		
PP39	PHL6	284	284	266	266	302	302	278	278	290	290	236	230	104	100	276	276	294	282	178	162	232	232	278	268		
PP40	PHL6	288	288	266	266	302	302	284	284	292	292	236	236	104	104	278	278	294	282	180	180	236	230	274	264		
PP47	PHL6	284	284	264	264	302	302	278	278	290	290	236	236	104	104	276	276	294	282	180	162	234	234	266	266		
NP03	PHL7	292	292	266	266	300	300	268	264	290	290	228	228	100	100	278	278	294	282	178	162	234	234	270	270		
NP06	PHL7	284	284	300	300	294	294	298	284	292	292	236	230	104	104	286	276	290	284	180	164	242	234	274	274		
NP08	PHL7	292	288	268	268	300	300	284	284	292	292	230	226	104	104	290	278	294	282	178	162	242	242	270	270		
NP10	PHL7	288	288	308	308	300	298	292	278	292	292	236	230	104	104	286	276	290	284	180	164	240	236	272	272		
NP15	PHL7	288	288	300	300	302	300	298	284	292	292	236	230	104	104	286	276	290	284	178	164	246	238	274	274		
NP20	PHL7	288	284	308	308	300	300	278	278	292	292	230	230	104	104	276	276	290	284	178	178	238	234	272	272		
MP04	PHL7	294	290	266	266	314	302	284	284	290	290	232	232	100	100	276	276	290	290	180	164	240	240	274	274		
MP06	PHL7	288	284	266	266	302	300	286	286	304	292	230	230	104	100	276	268	290	284	162	162	238	234	274	274		
MP08	PHL7	288	288	310	268	300	300	282	282	292	292	230	230	100	100	276	276	290	290	180	180	236	236	274	266		
SP04	PHL7	294	294	266	266	312	312	284	284	304	304	238	238	104	104	290	278	292	284	164	164	246	238	274	274		
SP06	PHL7	288	284	290	264	300	300	284	266	290	290	234	234	104	100	276	276	294	282	180	180	244	244	276	268		
SP12	PHL7	290	290	266	264	302	300	266	266	292	282	234	234	104	100	276	276	294	282	180	164	246	242	274	264		
SP15	PHL7	288	284	268	264	310	294	286	266	290	282	234	234	104	100	288	268	288	288	180	180	240	234	276	268		
SP18	PHL7	288	284	294	262	300	298	264	264	304	292	236	230	104	104	276	268	294	282	180	180	238	238	274	266		
SP24	PHL7	288	284	290	268	300	294	286	266	292	282	236	230	104	100	276	276	290	284	180	164	240	240	274	274		
